# Complex Wave Packet Dynamics Induced by Marangoni Stresses

**DOI:** 10.1021/acs.iecr.5c02378

**Published:** 2025-07-11

**Authors:** Ruofan Shi, Vignesh Thammanna Gurumurthy, Robert D. Tilton, Stephen Garoff

**Affiliations:** † Department of Chemical Engineering, 6612 Carnegie Mellon University, Pittsburgh, Pennsylvania 15213, United States; ‡ Department of Mechanical Engineering, Indian Institute of Technology Tirupati, Tirupati 517619, Andhra Pradesh, India; § Department of Biomedical Engineering, Carnegie Mellon University, Pittsburgh, Pennsylvania 15213, United States; ∥ Department of Physics, Carnegie Mellon University, Pittsburgh, Pennsylvania 15213, United States

## Abstract

New features emerge during Marangoni spreading of a surfactant-laden drop on a thick film, where inertia plays a significant role in the hydrodynamics relative to the more commonly studied low inertia spreading phenomenon. We uncover these features using high-speed imaging and understand their dynamics using numerical simulations on subphases of varying viscosity and depths. Deposition of a drop of surfactant solution drives the formation of a packet of waves moving across the surface. Waves closest to the deposition point are partially covered with the surfactant layer and are directly affected by Marangoni stresses; waves at larger distances are not. A previously unobserved event, merging of peaks close to the deposition point, is detected at early times in experiments and replicated in simulation. Such an event does not occur if a pure fluid drop of the same composition as the subphase is deposited on the surface. At later times, the difference in speed between the surfactant front and the innermost wave causes splitting of the innermost wave, creating a wave covered with surfactant and another wave not covered with surfactant. These features are detectable for thicker subphases of low viscosity liquids, commonly found in laboratory studies and technological settings where Marangoni spreading is present.

## Graphical abstract

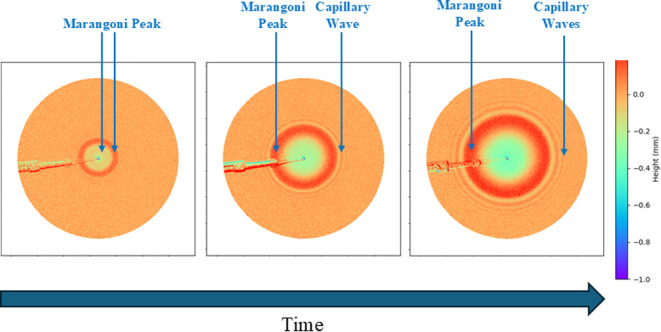

## 1 Introduction

Nonuniform distributions of surfactants at a fluid–fluid interface create surface tension gradients which induce tangential stresses at the interface. These stresses cause flows, known as solutal Marangoni flows, which proceed from low surface tension to high surface tension regions. A common example of this phenomenon is the localized deposition of surfactant on the interface, also referred to as Marangoni spreading. This flow plays key roles in applications such as pulmonary drug transport,^1–8^ deformation or cratering in coating films,^9–13^ Marangoni propulsion^14^ and “herding” of oil spills.^15,16^ In solutal Marangoni flows, the sharp surface concentration gradient at the surfactant front (the boundary between the surfactant-coated and surfactant-free regions) moving across the interface causes a localized surface tension gradient which then gives rise to a surface deformation that is sometimes referred to as the “Marangoni ridge”.^17–19^ This type of spreading has been studied previously for various surfactant and fluid systems, see for example refs 19–22 and references therein. When inertia is negligible, the Marangoni ridge is the dominant surface deformation feature found in experiments and modeling. Recent work has shown that under conditions for which inertia is significant, in addition to the Marangoni ridge, the impulse produced by sudden imposition of a surface tension gradient launches a group of gravity/capillary waves with amplitudes dependent on the depth and viscosity of the subphase.^23^ Hereafter, we refer to this group of waves as a wave packet.

Most of the literature on Marangoni spreading induced by deposition of surfactant laden drops has been limited to the case of high viscosity fluids on thin subphases where the Reynolds number is small, so inertia may be neglected, and the aspect ratio of film depth to spreading area is small, so the lubrication approximation may be invoked.^20,24,25^ Although a short-lived wave train ahead of the Marangoni ridge has been detected in simulation within the lubrication approximation,^26^ persistent capillary waves are suppressed in this high-viscous-thin subphase limit, and there is only a Marangoni ridge. Numerous theoretical^21,22,24–37^ and experimental^1,7,8,27,35,36,38,39^ studies in this regime are reported in the literature where the dynamics of surfactant front evolution and the ridge height are characterized. Experimental studies have characterized the speed and height of surface distortions traveling near the surfactant front as well as the impact of subphase depth, the magnitude of the surface tension difference between the surfactant drop and the clean subphase (represented by the “spreading parameter”), the surfactant deposition method, and the presence of pre-existing surfactant monolayers on the subphase.^2–4,40–43^

A key consequence of working on systems with higher viscosity and low aspect ratio is that capillary waves are suppressed.^44^ Work by Sauleda and Hsieh and co-workers explores Marangoni spreading on deep, lower viscosity subphases where lubrication theory no longer holds and inertia is important.^6^ Using simulation and experiment, they show both the Marangoni ridge as well as capillary waves moving across the surface are caused by the deposition of drops of a wide variety of soluble and insoluble surfactants. Their simulations reveal a new feature of the Marangoni spreading not reported previously: the formation of a new peak during the spreading, formed from the trailing edge of the Marangoni ridge when it splits into two peaks. Such a feature is unique to systems with non-negligible inertia.

Examining solutal Marangoni spreading in systems with non-negligible inertia is particularly important in probing the origins of the spreading behavior for the very common system when drops of surfactant solutions are deposited on aqueous subphases. For such systems, dewetting occurs for subphases less than ∼100 μm in thickness;^4^ moving contact lines and fingering behavior occur and fundamentally alter the spreading.^45–48^ Given the low viscosity of water and this limit on subphase thickness to maintain the subphase intact, solutal Marangoni spreading on water subphases must be treated in the regime where inertia is non-negligible.

The present work combines high speed imaging experiments and new simulations that provide more detailed insight into the driving forces and early time evolution of the wave packet in inertial systems, without being constrained by the assumptions underlying the lubrication limit. Within the wave packet created by the drop deposition, we observe waves with surfaces that are partially coated with surfactant and waves that have no surfactant on their surfaces. We show the waves without surfactant at their surfaces behave just as they would if there was no surfactant in the drop launching the wave packet. A new event at early times not previously reported is seen in both experiment and simulation and is the merging of two distinct surfactant-coated peaks into a single peak. With simulation, we further probe how the lagging of the surfactant front, which creates the splitting event reported in Sauleda and Hsieh et al.,^6^ is affected by the viscosity and thickness of the subphase.

In the following, we first give a general overview of the structure and evolution of the wave packet using simulation for a surfactant system with a Langmuir isotherm and simple one-specie adsorption kinetics. Next, we show strong similarities between experimental observations and simulations, thus justifying the use of the simulation. We then use simulation to probe the spreading for a simple, one specie surfactant system, not to try to match quantitatively the behavior of the one specific surfactant system used in the experiments but to probe the spreading where the surfactant behavior is simple. Using these simulations, we provide more details about the wave packet behavior with emphasis on the newly discovered merging event and the splitting event, and we discuss how key spreading features depend on whether adsorption is diffusion-limited or reaction-limited. Finally, we discuss the effect of subphase thickness and liquid viscosity on the spreading and use these results to show how the spreading behavior changes as the viscosity is increased and the subphase thickness is decreased but still remaining outside the lubrication approximation regime.

## 2 Experiments and Numerical Modeling

### 2.1 Materials and Material Systems

The water-soluble surfactant sodium dodecyl sulfate (SDS) (Sigma-Aldrich, ≥99%, CAT#436143) was used as received. All water was purified by a Milli-Q (eq 7000) water treatment system. Glycerol (Sigma-Aldrich, ≥99%, CAT#G7757) was used to increase subphase viscosity. All subphases contain 0.01 g/L erythrosine B dye (Sigma-Aldrich, >80%, CAT#E8886) in support of the absorbance-based imaging technique. Talc tracer particles (Fisher Scientific, CAT#T2–500) were dispersed on the surface of the liquid subphase to track surface flows during some experiments. A Corning glass Petri dish with a 14.5 cm diameter was used for all spreading experiments. The Petri dish was rinsed with ethanol (Pharmco, >99.5%, CAT#111000200) and purified water before and after experiments.

A variety of systems were examined in experiment and simulation to probe the evolution of the wave packets as Marangoni and viscous stresses vary. Supporting Information (SI) Tables S1 (experiments), S2 (simulation), and S3 (simulation) present all the systems examined. None of the systems examined in experiment or simulation showed dewetting of the substrate; and all were in the central depression regime.^4^ In experiments, the fluid in the drop and subphase were glycerol/water mixtures with the same glycerol/water content in the drop and the subphase. The concentration of glycerol in the solution was changed to vary the viscosity of the subphase and drop. The subphase thickness was also varied from 0.2 mm to 5 mm. The drop in all experiments contained 82 mM SDS and had a volume of 6.0 μL. This SDS concentration in the drop is ten times higher than its CMC.^49^ Experiments were conducted at 20 ± 1 °C. The dye concentration was 0.01g/L. At this dye concentration the surface tension of the drop is not significantly changed.^4^ This concentration is optimal for our optical detection described below, allowing all measured optical densities to be in the linear region of the Beer’s Law curve relating absorbance to subphase depth for our system.

### 2.2 Experimental Methods

Experiments were designed to measure the local height of the subphase as the wave packet moves across the surface. The experimental setup and methods, shown in Figure 1, were essentially the same as that used in prior work from this group^4,6^ and details may be found in those references. The key change from that work was that in the present work, a high frame rate camera was used. The experimental setup consists of a Petri dish containing the subphase fluid with dye, which is illuminated from below. The transmitted image is recorded by a camera located above the Petri dish, and the local intensity is used to calculate the local height via Beer’s Law. Calibration curves ensured experiments were conducted in the linear regime of the intensity response of the camera. A 500–550 nm bandpass filter with 525 nm center wavelength (Edmund Optics, CAT#86963) was mounted in front of the camera to allow only wavelengths in the main absorption peak of the erythrosine dye to be recorded. Restricting the detection to only those wavelengths within the band ensures that the absorbance response to subphase thickness changes is properly resolved. The surfactant solution drop was gently placed on the surface at the center of the dish via a micropipette with as little kinetic energy as possible.

**Figure 1:**
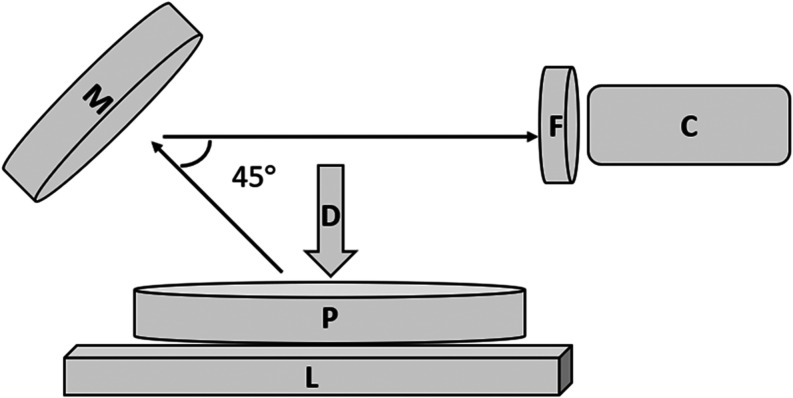
Experimental setup. A Petri dish (P) lays on top of a battery powered light pad (L). The drop deposition point (D) is at the center of the Petri dish. The spreading event is recorded via the high-speed camera (C). A mirror (M) oriented at 45 deg reflects the light into the camera. A bandpass filter (F) admits wavelengths matching the main absorbance peak of the subphase dye into the camera.

The illumination was produced by a battery powered light pad (ASRAS, Rechargeable A4 Light Box Tracer). A light-blocking screen was placed on the light pad with a hole the same size as the Petri dish to block stray light from entering the camera. A flat mirror was located above the Petri dish at an angle of 45° to reflect the image to the high-speed camera (Phantom, Phantom v 9.1). The image data was recorded by a Phantom Control System (Phantom, Phantom Cine Viewer, PCC 3.7). A frame rate of 300 frames per second with the maximum exposure time (0.0033 s) provided a sufficiently fast frame rate to record the early time behavior of the spreading with the highest possible signal-to-noise ratio. This ratio allowed the identification of the positions of the key surface distortions with time but did not allow the determination of the shapes of these distortions. Memory in the system allowed recording of 8 s of data, which was more than enough to record entire spreading events.

Image data collected from the camera control system were split by Phantom Cine Viewer (Phantom CV 3.7) into separate time frames and analyzed using ImageJ.^50^ Contrast of the image was varied to optimize the visibility of surface distortions and the positions of peaks were determined for all times during the spreading event.^51^

### 2.3 Simulation

Simulations were performed to identify trends in how the evolution of the waves depends on critical system parameters, namely the viscosity of the fluids and the thickness of the subphase. Simulations were also performed to check whether the size of the drop or the adsorption and desorption kinetics of the surfactant being reaction or diffusion limited would affect the general trends reported. In simulations, the surfactant in the drop was below the critical micelle concentration (CMC), to allow the use of a simple adsorption/desorption kinetic model without introducing additional unknown parameters for micelle transport and breakup. Therefore, in the simulation, the surface tension at the center of the drop immediately begins to increase as surfactant is depleted during spreading, whereas this was unlikely to occur in the experiments with surfactant concentrations that exceeded the CMC.

Simulations were carried out using the same equations and numerical methods as used in ref 6 and detailed in Section S2 of SI. Here, we provide a brief overview of the simulation methodology. The spreading is modeled by placing a drop of surfactant solution on the subphase and the hydrodynamics and surfactant transport are treated allowing material properties to be varied except for the shape of the initial drop and the assumption of a model of the adsorption kinetics. The model formulation includes the full Navier–Stokes equation and boundary conditions, a surfactant equation of state consistent with the chosen adsorption model, and mass transport of the surfactant that includes bulk and surface advection and diffusion. Simulations use the Langmuir adsorption model to capture key aspects of the phenomenon, in particular the smooth transition from linear, Henry’s law behavior to surface saturation with increasing surfactant concentration. The equations are written in cylindrical coordinates and solved by finite element methods using COMSOL 5.6. The radius of the computation domain was set so no waves were reflected from the boundaries in the time scale of the simulation. The computation was benchmarked by comparison to the predictions for a system that conforms to the lubrication approximation.^25^ Convergence was verified by changing the mesh size within the COMSOL Multiphysics module. Based on a grid independence study, we have chosen a mesh with 226,011 elements and a maximum cell size of 0.00738 cm. See Section S3 in SI for details.

To show that the results reported apply to a wide range of soluble surfactants, we simulate cases ranging from systems that are diffusion limited, with adsortion rate constant, *k*_a_ = 10,000 m^3^ mol^–1^ s^–1^ and Damkohler number of the second kind (*DaII*, adsorption rate/diffusive transport to the interface)^51^ *DaII* = 3200, to systems that are reaction limited, with *k*_a_ = 0.1 m^3^ mol^–1^ s^–1^ and *DaII* = 0.032 (see Table S3). Our base case with *k*_a_ *=* 1000 m^3^ mol^–1^ s^–1^ and *DaII* = 320 (see Table S2 line 2) is in between these limits. Since speeds in Marangoni spreading on deep subphases are typically *U* ∼ 0.1 m/s,^5,6^ (producing a surface Peclet number of ∼10^6^) the Damkohler number of the first kind (*DaI*, adsorption rate/rate of lateral convection = *k*_a_ *C*_o_ *L*/*U*) ranges from ∼20 to 2 × 10^6^ for the same range of *k*_a_ values, using the typical bulk concentration used in the simulation (*C*_o_ = 0.2 mol/m^3^) and *L* = 0.1 m as the length scale of the lateral flow. Thus, the adsorption rate is an order of magnitude to 6 orders of magnitude greater than the rate of convective transport along the surface, whereby the details of the adsorption kinetic model and choice of *k*_a_ are not very important in the simulations. Other parameters describing the surfactant isotherms were chosen to adequately represent simple, monomeric surfactants.^5^

## 3 Results and Discussion

### 3.1 General Description of the Spreading

Here we present an overview of the entire spreading event. In Section 3.3, we will discuss each feature of the spreading in more detail. Figure 2 shows simulation results for the interface shape, surfactant surface excess concentration and flow fields during the spreading event, and Figure 3 shows the position vs time for all peaks and the surfactant front. (The next section will show comparisons between experiments and simulation that justify the use of the simulation). To show the relationship between the interface shapes and flow fields and the underlying surface tensions and surface tension gradients, Figures S2 and S3 in SI show interface shapes, superimposed on plots of surface tensions and surface tension gradients. Figure S3 in SI shows that the driving force for spreading, the surfactant gradient, is strongly peaked at the surfactant front.

**Figure 2:**
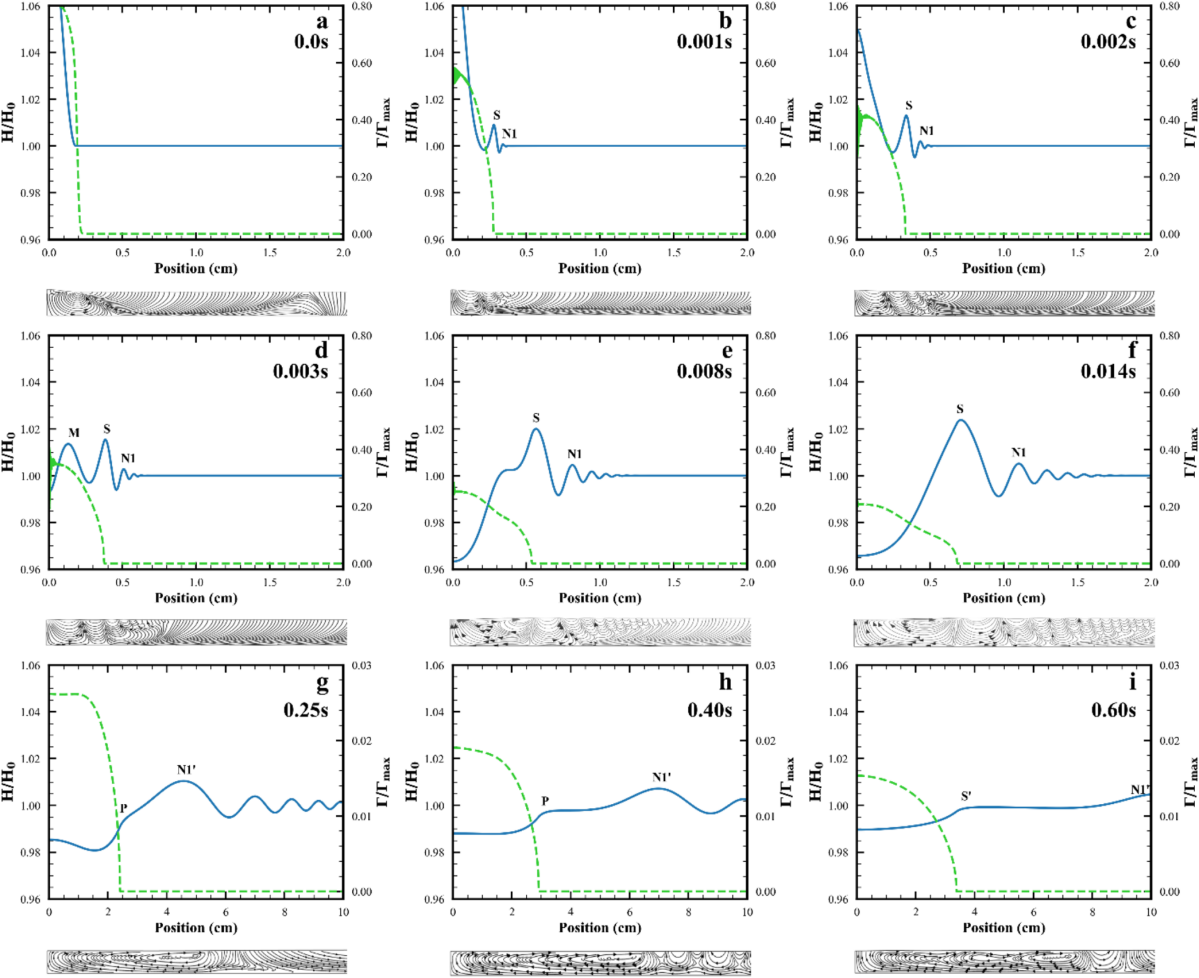
Spreading event for base case: soluble surfactant spreading on a 2.4 mm deep, 1 mPa·s viscosity subphase (line 2 in Table S2 and base case in Table S3). Figures include interface shape, blue solid line; surfactant surface excess concentration, green dashed line; and flow field in black. Panels (a–c) illustrate the relaxation of the deposited drop for (a) the initial condition, (b) *t* = 0.001 s, (c) *t* = 0.002 s. Panels (d–f) show the merging event with (d) the merging peak, M, and the S peak before merging at *t* = 0.003 s, (e) the merging peak has been reduced to an inflection point during merging at *t* = 0.008 s, and (f) the S peak after merging at *t* = 0.014 s. Panels (g–i) show the subsequent splitting event with (g) showing the S peak has been reduced to a the shoulder on the N1 peak at *t* = 0.25 s, (h) the shoulder has become stronger and is an inflection point in the interface shape at *t* = 0.40 s, and (i) the inflection has developed into a maximum marking a new peak S′ by *t* = 0.6 s. The flow field plots illustrate the velocity underneath the fluid surface. High frequency oscillations near *r* = 0 at early times on surfactant surface excess arise from the shape used for the drop in the initial conditions (see eq 2 in SI) and have no effect on any behaviors discussed.

**Figure 3:**
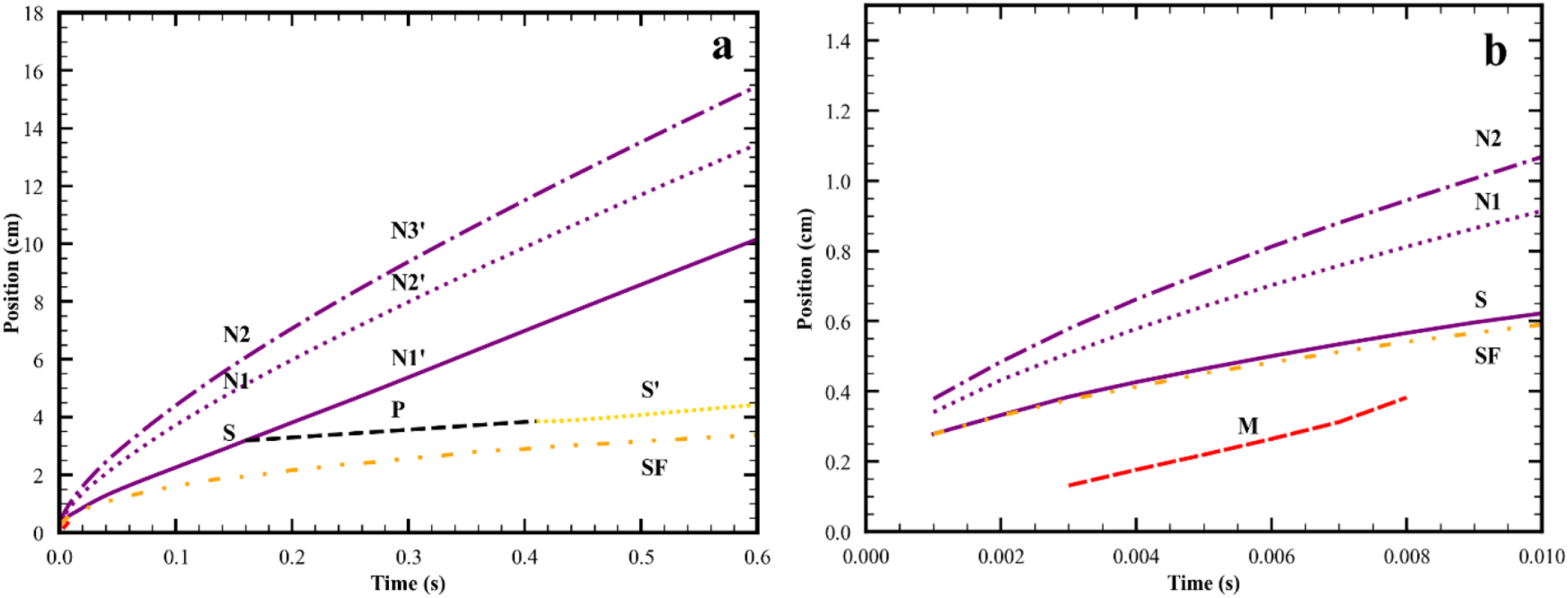
Time dependence of the radial positions of the surfactant front SF (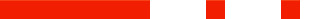), M­(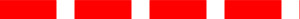), P­(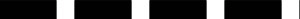), S′(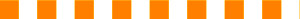), and the first (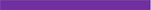), second (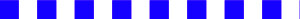), and third (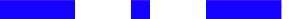) peaks in the wave packet. (a) entire spreading event. (b) only early times during merging event. Curves are labeled to indicate their role in the spreading event. At early times before splitting starts: M is the merging peak, S is the peak in the vacinity of the surfactant front, N1 and N2 are peaks not in the vacinity of the surfactant front. During splitting: P is the plateau formed from the S peak; N1′, N2′ and N3′ are peaks not in the vacinity of the surfactant front. After completion of the splitting: S′ is the peak in the vacinity of the surfactant front; N1′, N2′ and N3′ are peaks not in the vacinity of the surfactant front. Parameters for simulation in line 2 of Table S2.

#### 3.1.1 Drop Relaxation

As seen in Figure 2a through 2c, the deposited drop completes its relaxation (defined as occurring when the interface height at the center, *r* = 0, crosses the undisturbed surface height) between 0.002 and 0.003 s and this relaxation drives the formation of the wave packet that propagates across the surface. In Figure 2b (0.001 s), a peak has formed (marked S) with a maximum just ahead of the surfactant front and which is heavily influenced by Marangoni stresses and a second peak (marked N1) which is not covered by surfactant and not influenced by Marangoni stresses. In Figure 2d (*t* = 0.003 s), immediately after relaxation of the deposited drop, a new peak has formed (marked M) which was not present earlier; and the two peaks (M and S) at the positions closest to the deposition point have surfactant on their surfaces. This is seen by comparing the surfactant surface excess concentration (green) and surface height (blue) plots and in Figure 3 which shows the position of these peaks and the surfactant front vs time.

At *t* = 0.003 s, only the M and S waves are directly affected by the Marangoni stresses arising from the surfactant concentration gradient. The flow field under these waves and later waves directly affected by Marangoni stresses shows similarities to structures seen below Marangoni waves in the lubrication approximation (i.e., a recirculating flow under the waves and an imperfect alignment of the peaks and troughs of the surface deformations with the locations where the flow is toward or away from the surface).^25,52,53^ The peaks at larger radial distances (with the first marked N1) do not have surfactant on their surfaces and thus are not directly affected by Marangoni stresses. The heights of these waves decay with increasing radial distance. The flow field for these waves is typical of those under simple capillary waves in cases with no surfactant deposited (i.e., the locations where the flow directly impinges on the surface align with peaks and where the flow is moving directly away from the surface align with troughs).^23^

#### 3.1.2 Key Event 1: Peak Merging

Two key events occur during the spreading event. Figure 2d–f show the first (“merging”) event is a combining of the two peaks at the smallest radial positions and which have surfactant on their surfaces. This merging event, which has not been predicted theoretically or observed experimentally before, is seen in experiments as will be discussed in Section 3.2. Before merging, in Figure 2d, the two innermost peaks (M, S) are clearly separated from one another. During merging, in Figure 2e, the merging peak, M, is close enough to the S peak that only an inflection point remains. As the merging event ends, in Figure 2f, only one peak (S) with surfactant on its surface remains with a slight bump on the small *r* side of that peak, the remnant of the merging peak.

#### 3.1.3 Key Event 2: Peak Splitting

The second event, termed splitting, was first identified in ref 6. As seen in Figure 3, the surfactant front is always moving slower than the S peak. Figure 2g and h show that as the surfactant front lags behind the S peak, a plateau is formed. The position of the small r boundary of the plateau versus time is marked P in Figure 3. Finally at *t* = 0.41 s, a new maximum, and thus a new peak partially covered with surfactant, S′, is formed. From the time the plateau begins to form, the innermost peak now has no surfactant on its surface and so is now called N1′.

To place all these these results in the perspective of the lubrication approximation, we compare the general features seen in the base case above with simulations that are within the lubrication approximation (see Figure S4 in SI). When the initial subphase height is 0.2 mm, the viscosity is 1410 mPa·s, the aspect ratio is 0.11, and the Reynolds number is 4.4 × 10^–4^, conditions fall within the lubrication regime (line 11 in Table S2). All capillary waves are suppressed and only one peak remains, and this peak has surfactant on its surface. The M peak that is formed in the high Re case never forms, so the merging event is not present. The surfactant front does not fall behind the remaining peak, so no splitting events occur. These behaviors are consistent with previously published results for the lubrication regime which examine a wide variety of systems.^20,26,54,55^

### 3.2 Experimental Results

Drawing fundamental conclusions from the simulation results is justified by the matching of three key features of the structure and evolution of the wave packet in both experiments and simulations: (1) the occurrence of the early time merging event, (2) the tracking of the surfactant front relative to the peak on the interface during and after the merging event, and (3) the evolution of the peak positions vs time are very similar for the experiments and simulation. This is the primary purpose of the experiments, to support the validity of key phenomena predicted by the numerical simulations, rather than to serve as a testbed for model fitting.

The early time merging event described above occurs in both experiment and simulation. Figure 4 shows experimental images. In Figure 4a, the S peak has formed but the M peak is not yet detected. In Figure 4b,c, the M and S peaks are both detectable with the M peak moving closer to the S peak. Finally in Figure 4d, the M peak has disappeared and the at this point the N1 peak becomes visible. These results are typical of the early time behavior seen in the various experimental systems listed in Table S1 They are entirely consistent with the merging event in the simulation results in Figure 2c–f. Details of the merging behavior and its origins will be discussed later in Section 3.3.2, but the similarity of the experimental and simulation results strongly suggests the simulation is capturing the essential behavior of this previously unreported event. While the merging event is observed consistently in repeated experiments, the experiments are not sensitive enough to low amplitude waves to capture the splitting event predicted in the simulation (Figure 2g–i).

**Figure 4:**
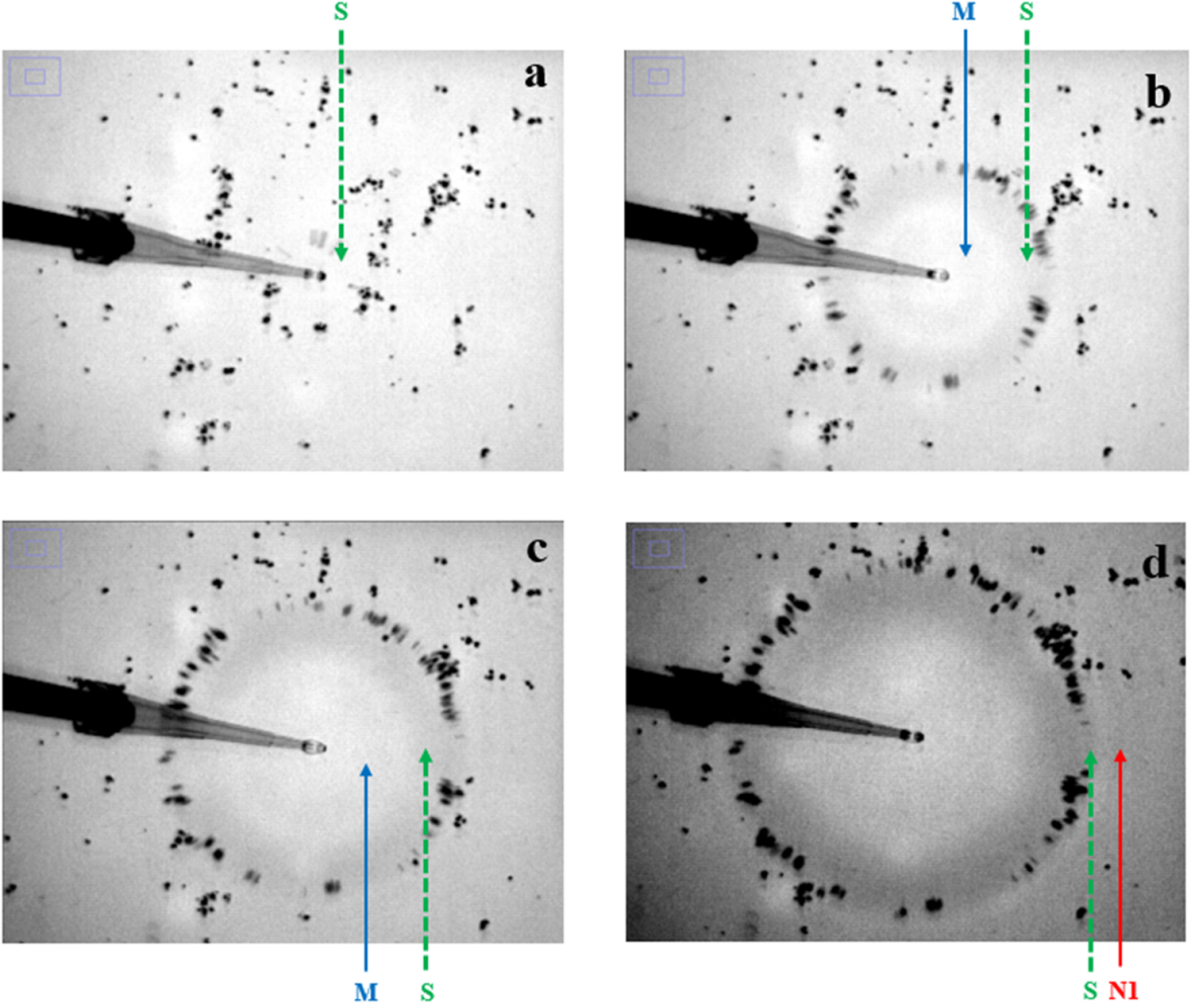
Experimental images of spreading event for a typical experiment. Merging event shown. (a) *t* = 0.003 s. (b) *t* = 0.02s. (c) *t* = 0.03 s. (d) *t* = 0.04 s. Black dots are talc particles. Dark rings are peaks in the surface deformation and bright areas are toughs. Blue solid arrow points to merging peak, M. Green dashed arrow points to surfactant covered peak, S. Red arrow points to peak with no surfactant, N1. Peaks M and S have merged by panel (d). The N1 peak is only visible at the later time in these particular images. Parameters for experiment listed on line 1 Table S1.

The second similar feature found in both experiments and simulations is the close proximity of the surfactant front, marked by the inner boundary of the talc particles,^6^ to the maximum of the S peak during the merging event. In both experiment (Figure 4) and simulation (Figure 2d–f), the surfactant front is very close to the S peak throughout the merging event. This tracking is observed in all the systems examined with simulation in Table S2 and experiments in Table S1 when talc was deposited on the surface to track the surfactant front.

Finally, all peak positions vs time are strikingly similar throughout the entire spreading event in both experimental and simulation results as shown in Figure 5. In Figure S5 of SI, data from other experimental conditions are shown to also be very similar to the simulation and the experimental base case shown in Figure 5. Power laws have been predicted for the time dependence of the surfactant fronts within the lubrication approximation (see for example ref 26), at high Reynolds number on thicker films^54^, and for the related problem of a spreading drop of pure liquid (see for example ref 56). Attempted power law fits to both the experimental S peak, which follows the surfactant front at early times, and the surfactant front in simulations show statistically significant deviations from power laws; nevertheless, the data are approximated by powers ∼0.6 for the experimental data and ∼0.4 for the simulation (see the insets in Figure 5). A power of 3/8 has been predicted for the movement of the surfactant front of an insoluble monolayer deposited on a deep fluid pool.^54^

**Figure 5:**
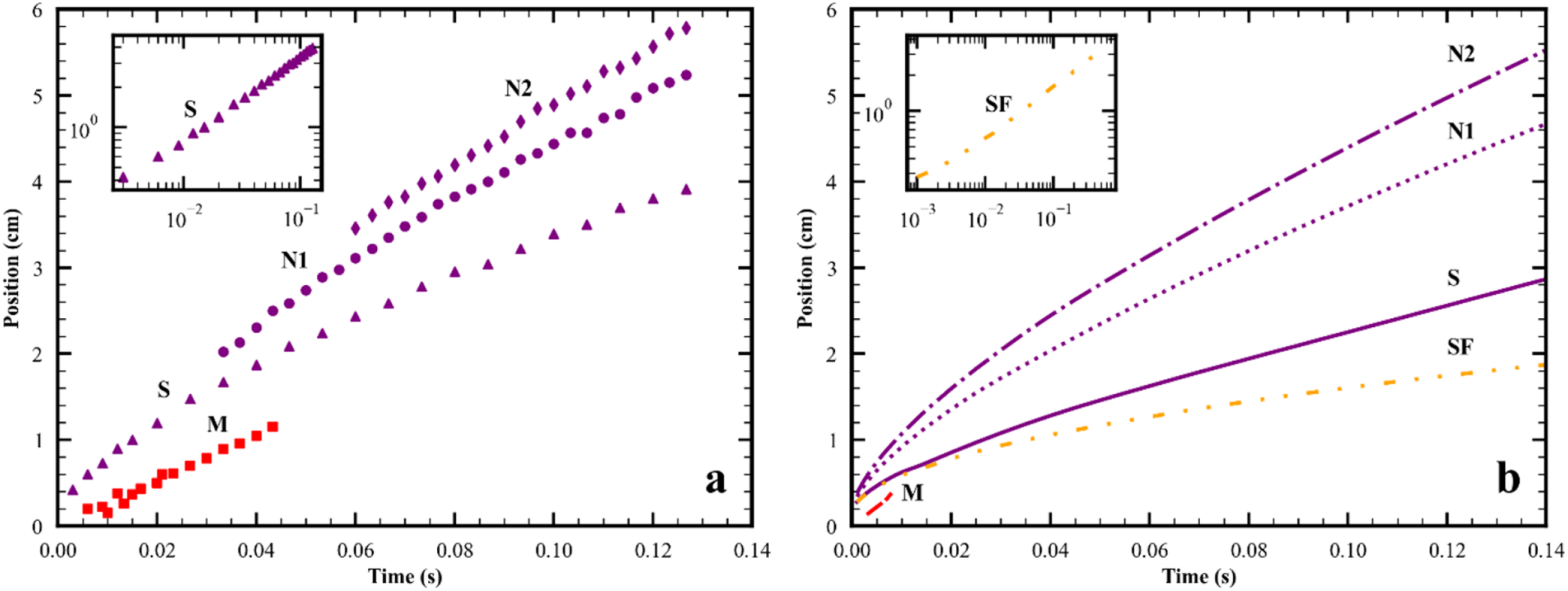
Time dependence of the radial positions of the surfactant front and peaks in the wave packet. (a) Experimental data; inset log/log plot of S. (Red solid box) M, (purple solid triangle) first peak, (purple solid circle) second peak, (purple solid diamond) third peak. (b) Simulation results; inset log/log plot of SF. (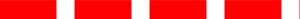) M, (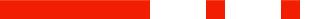) surfactant front, (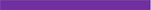) S, (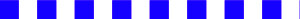) N1, (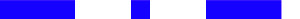) N2.

### 3.3 Marangoni Stress Effects on Different Portions of the Wave Packet

Having established consistency between experiments and key predictions of the numerical simulation that validate the simulation, the remainder of the results and discussion will be based on the simulations.

#### 3.3.1 Behavior of Waves without Surfactant on Their Surfaces

Without surfactant in the drop (drop and subphase surface tension = 72.5 mN/m), gravity, viscosity, and surface tension contribute to the relaxation of the distortion of the fluid–fluid interface due to the coalescence of the initial drop with the surface. The simulation shows that the presence of the surfactant in the drop causes Marangoni stresses along the drop/air interface (see Figure 2) and therefore will cause an additional stress relaxing the drop. Since the momentum imparted to the fluid by this initial relaxation creates the wave packet, the wave packets created with or without surfactant in the initial drop should differ from one another. Figure 6a compares wave packets produced with or without surfactant in the initial drop at 0.001 s, which is before the merging peak even appears when the drop contains surfactant. Figure 6b compares wave packets produced with or without surfactant in the initial drop at 0.003 s, which is before the merging event occurs when the drop contains surfactant. Figure 6c shows the same comparison for a later time, which is after the merging event and at the onset of the splitting event that only occurs for surfactant-laden drop deposition. The number of peaks and the height of the peaks are increased when surfactant is present in the drop compared to the case of surfactant-free drop.

**Figure 6:**
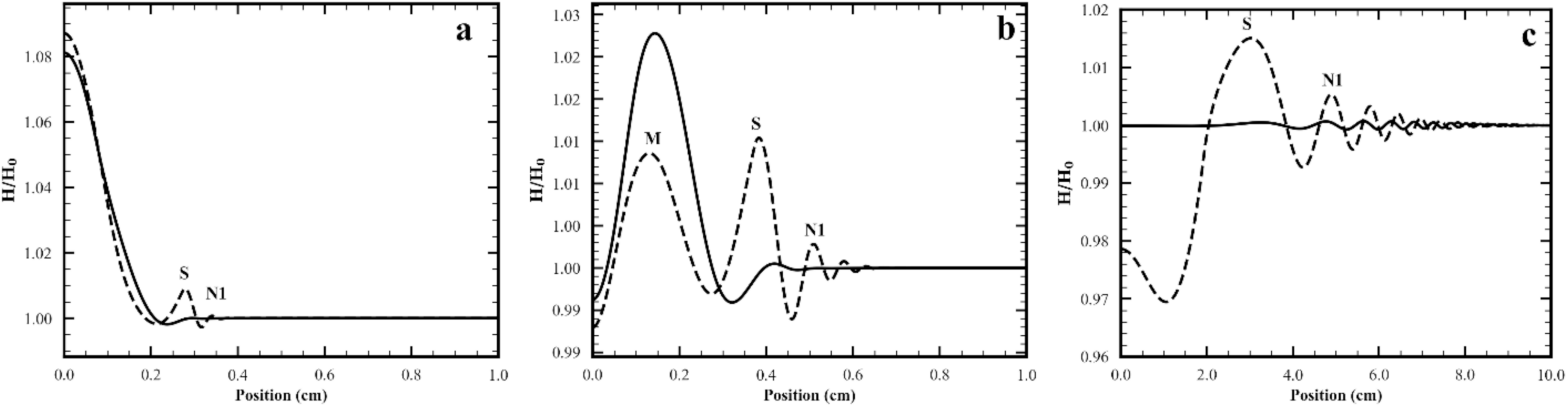
Comparison of wave packets arising without (solid line) or with surfactant (dotted line) in the initial drop. (a) *t* = 0.001 s. (b) *t* = 0.003 s. (c) *t* = 0.15 s. Parameters for simulation listed on line 2 Table S2 and base case in Table S3.

The merging event involves waves that have surfactants on their surfaces. While this is unique to deposition of surfactant-laden drops, both surfactant-free and surfactant-laden drops launch a packet of waves that never have surfactant on their surfaces. Figure 7 shows that those peaks which do not have surfactant on their surfaces have the same dispersion curve, regardless of whether there is surfactant in the deposited drop or not. Furthermore, this data is well modeled by the dispersion curve for waves launched by a drop with no surfactant on a subphase of the same depth and surface tension (72.5 mN/m) (line 2 Table S2) used in the simulation of a drop with surfactant.^23^ Therefore, at the higher Reynolds numbers in the present study, the existence of Marangoni stresses at smaller radial positions has negligible far-field effects on the propagation of the waves that exist at larger radial position in the wave packet launched by a surfactant-laden drop but have no surfactant on their surfaces.

**Figure 7:**
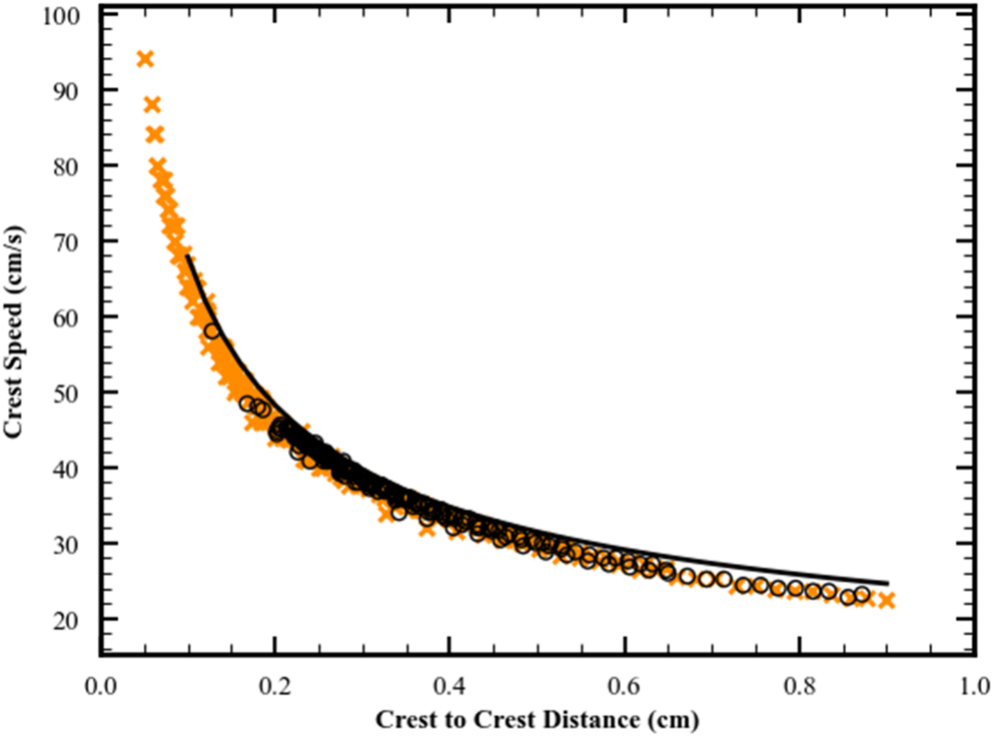
Dispersion curves for waves that are not covered by surfactant when the waves are launched by deposition of a surfactant-laden drop (orange x) or a surfactant-free drop (black O). Using simulations for drops with and without surfactant, crest to crest distances were found from interface shape data at a series of times and wave velocities were determined by numerical differentiation of the position vs time data for each peak present in the wave packets. Theoretical dispersion curve (solid line) is for a surfactant-free system (subphase surface tension 72.5 mN/m and depth 2.4 mm) and includes the correction for the finite thickness of a liquid film.^23^

#### 3.3.2 Behavior of Waves with Surfactant on Their Surfaces: Merging and Splitting Events

Figure 2a–c show that as the deposited drop relaxes, a wave (S) is driven outward with surfactant covering part of its surface and the location of its maximum very close to the surfactant front. This peak is formed outside the initial drop radius. As the surface height at *r* = 0 decreases to the initial unperturbed subphase height (between Figure 2c and d), a peak at smaller *r* is formed, and this peak (M) is entirely covered by surfactant. Comparing Figure 2c and d shows that this merging peak forms inside the initial drop radius and so represents a deformation of the drop surface. As seen in Figure 3b, it moves faster than S, which was formed first. This larger speed may arise because the surface tension gradient extends across the entire surface of the merging peak, while the S peak is only half covered by a surfactant gradient, although the gradient across S is larger than across M (see Figure S3 in SI).

As the merging event continues (Figure 2d through 2f), the composite of the two peaks evolves from two distinct peaks to only an inflection point, and finally to a single asymmetrical peak. The flow under the peaks involved in this event evolves from two distinct, oppositely directed recirculation flows under the merging peak and one recirculation flow under the S1 peak at larger *r* to only two recirculation flows associated with the newly synthesized peak. This scenario for the merging event is maintained even when the initial drop size is varied (see Figure S6 in SI) or the surfactant adsorption kinetics is varied from diffusion limited to adsorption limited (see Figure S7 in SI). As seen in Figure S8 in SI, with no surfactant in the deposited drop, no merging occurs. Peaks emerge from the relaxation of the deposited drop and simply propagate outward as part of the wave packet created by the relaxation of the drop.

As the merging event ends at *t* ≈ 0.014 s (see Figure 2f), the surfactant front and the largest surface tension gradient (which is always associated with the surfactant front) are located just behind the peak of the surfactant covered wave, S. As shown in Figure 3 when the merging ends, the surfactant front is moving more slowly than the S peak causing the surfactant front to increasingly lag behind the maximum in the S peak. This lagging of the surfactant front relative to the S peak causes the second key event, the splitting, to occur. As the surfactant front lags, the shoulder on the S peak becomes more prominent (see Figure 2g) and then becomes a plateau,with the surfactant front located at the small r boundary of the plateau (see Figure 2h). Figure 8 shows this transition as it occurs at times between those of Figure 2g and i; and on Figure 3, the location of the shoulder and small r boundary of the plateau are approximately indicated by the dashed line. During this time, there is no peak that is covered with surfactant, and what was formerly S has become the innermost peak with no surfactant on its surface, the new N peak (denoted N1′ to distinguish it from the original N1 peak that had no surfactant on its surface). The flow field at the location of N1′ is now similar to the flow under peaks that have no direct influence from Marnagoni stresses. At *t* = 0.41 s a maximum (a new peak) develops on the small r edge of the plateau. The surfactant front is in close proximity to this maximum so it is influenced by Marangoni stresses. Therefore, it is marked denoted S′ The flow field at the location of S′ is now similar to that under the innermost peak with surfactant on its surface at earlier times. This scenario for the splitting event is maintained when the surfactant adsorption kinetics is varied from diffusion limited to adsorption limited (see Figure S7 in SI).

**Figure 8:**
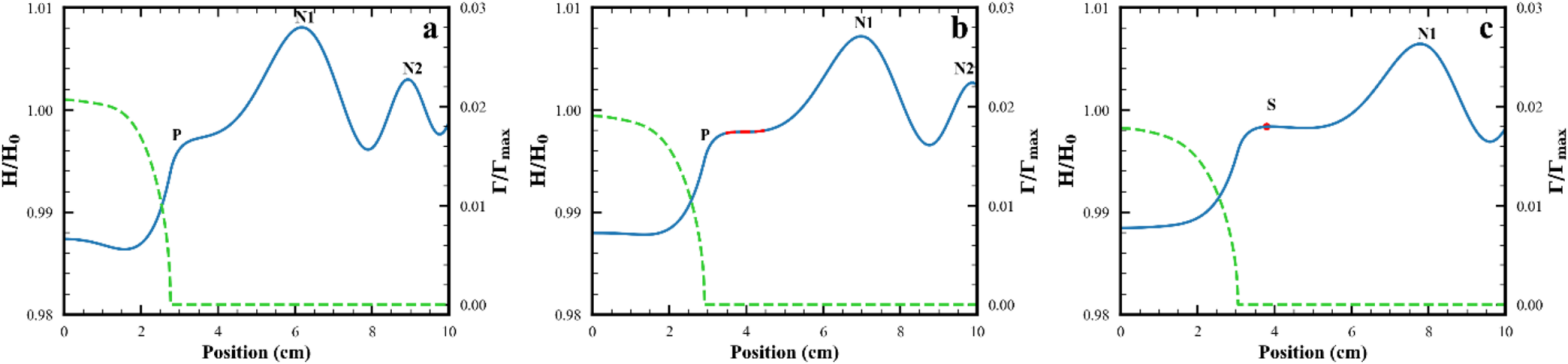
Surface height evolution through the splitting event. Figures include interface shape, blue solid line; surfactant surface excess concentration, green dashed line. (a) *t* = 0.35 s where plateau has formed. (b) *t* = 0.40 s where an inflection point has formed on the interface and is marked by the red line. (c) *t* = 0.45 s where a maximum has formed on the interface and is marked by the red dot. Refer to Figure 2g–i for full view of surface shapes, surfactant front and flow fields during splitting. Parameters for simulation given on line 2 of Table S2 and base case in Table S3.

As seen in Figure 3, after the splitting event has concluded (*t* = 0.41 s), the surfactant front is still traveling more slowly than the S′ peak. Since this growing lag between the surfactant front and the peak was the precursor state to the splitting event, this lag could potentially set the stage for another splitting event at later times. This was not observed for any of the cases simulated. It is noted that the speed difference between the surfactant front and S peak, 8.2 cm/s entering the splitting event at *t* = 0.2 s, is much larger than the speed difference between the surfactant front and the S′ peak, 0.32 cm/s, exiting the splitting event here at *t* = 0.5 s. So another splitting event either may be long delayed or may even not occur since peak heights, which decrease with time, may become negligible at such a late time.

### 3.4 Effect of Increasing Viscosity and Decreasing Subphase Thickness on Wave Packet

As the viscosity of the subphase and drop are increased (maintaining the condition that the subphase and drop viscosities are equal), and the initial thickness of the subphase is decreased, the Reynolds number *Re* and aspect ratio ε decrease. The system then approaches the lubrication approximation. As seen in Table S2, using simulation, the impact of increasing viscosity (from 1 to 60 mPa·s) at fixed subphase thickness (2.4 mm) and decreasing thickness (from 5 to 1 mm) at fixed viscosity (1 mPa·s) have been probed while remaining in the regime where the lubrication approximation does not yet hold (*Re* > 20, ε > 0.5). Spreading changes are due to increases in the viscous stresses throughout the subphase associated with a larger viscosity and for thinner subphases are due to placing the location of the no slip boundary condition on the bottom surface of the subphase closer to the free surface where the Marangoni stresses act. If the viscosity is raised to 1410 mPa·s and the subphase thickness is decreased to 0.2 mm, the lubrication approximation holds (*Re* = 0.004 and ε = 0.11). In this limit the capillary waves become insignificant and only one wave, partially covered by surfactant, remains (see Figure S4 in SI). Since the time scaling normally used in the lubrication approximation (where the characteristic time is $t_{c}=\frac{\mu R_{0}^{2}}{SH_{0}}$ ^25^) does not collapse any of the position vs time data for the systems that are outside the lubrication approximation regime (see Figure S9 in SI), systems are compared in real time.

#### 3.4.1 Effects on Waves without Surfactant on Their Surfaces

As seen in Figures S10 and S11 in SI, the number of waves without surfactant on their surfaces in the wave packet decreases as the viscosity is increased at fixed subphase thickness, as would be expected since increasing the viscosity of the relaxing drop reduces the kinetic energy imparted to the waves launched by that drop relaxation. The amplitude of the waves without surfactant on their surfaces is also decreased. The trends for decreasing initial subphase thickness at fixed viscosity on the waves without surfactant on their surface are qualitatively the same but smaller in magnitude for the factor of 5 variation in thicknesses examined (see Figure S12 in SI). The waves with no surfactant on their interface disappear when the lubrication approximation condition is reached (see Figure S4 in SI). The speeds of all the waves without surfactant at their surfaces decrease for increasing viscosity and decreased thickness as predicted for simple capillary waves on dissipative subphases^57^ and finite thickness.^23^ As shown in Figures S13 and S14 in SI, these results from simulation are consistent with experimental data obtained for the systems in lines 1, 4 to 7 in Table S1 in SI.

#### 3.4.2 Effects on Waves with Surfactant on Their Surfaces: Merging and Splitting Events

With increasing viscosity (1 to 60 mPa·s) or decreasing subphase thickness (5 to 1 mm), simulation shows that the speeds of the surfactant front and of the wave with surfactant on its surface, S, both decrease, as summarized in Figures 9 and 10. The amplitude of the S wave decreases as seen in Figures S10–S12 in SI.

**Figure 9:**
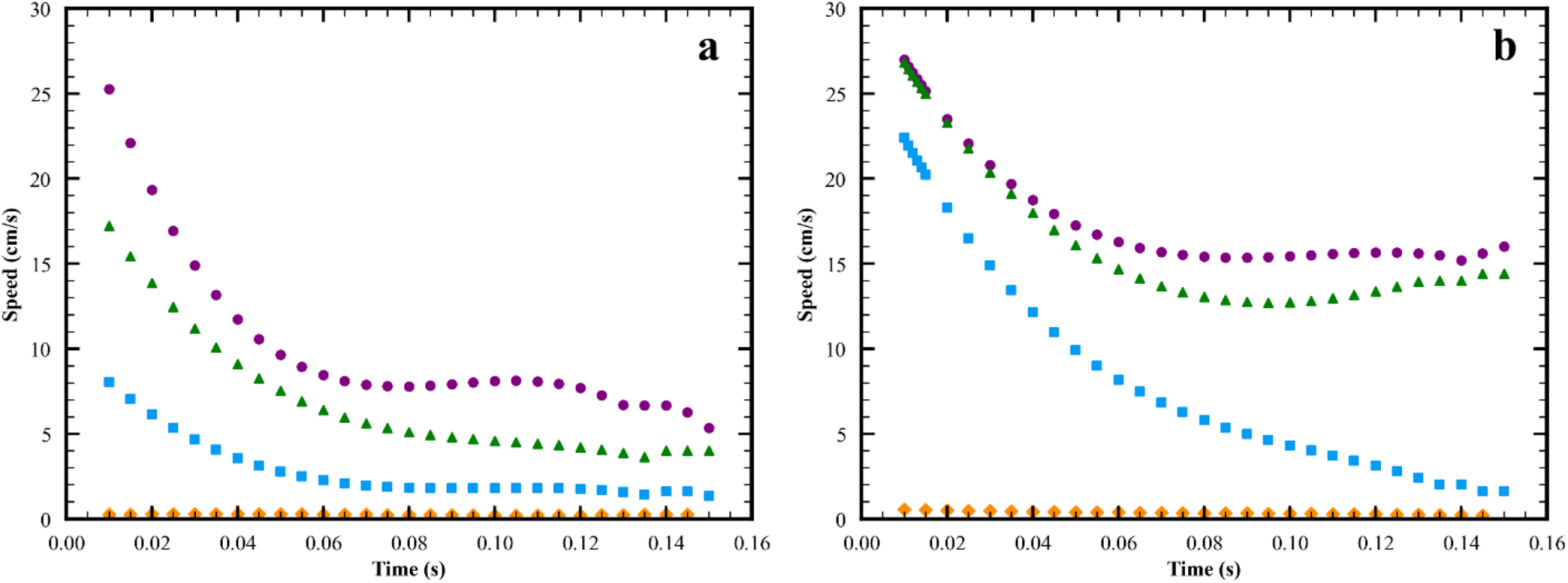
Changes in speed of surfactant front and S1 wave with viscosity at subphase thickness of 2.4 mm. (a) Surfactant front. (b) S1 peak. (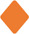, lubrication approximation; 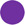, 1 mPa·s; 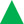, 6 mPa·s; 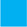, 60 mPa·s). In (a), the speed of the lubrication approximation data ranges from 0.22 to 0.26 cm/s. In (b), the speed of the lubrication approximation data ranges from 0.56 to 0.19 cm/s. Parameters in lines 2, 7, 8, 11 in Table S2 and base case in Table S3.

**Figure 10:**
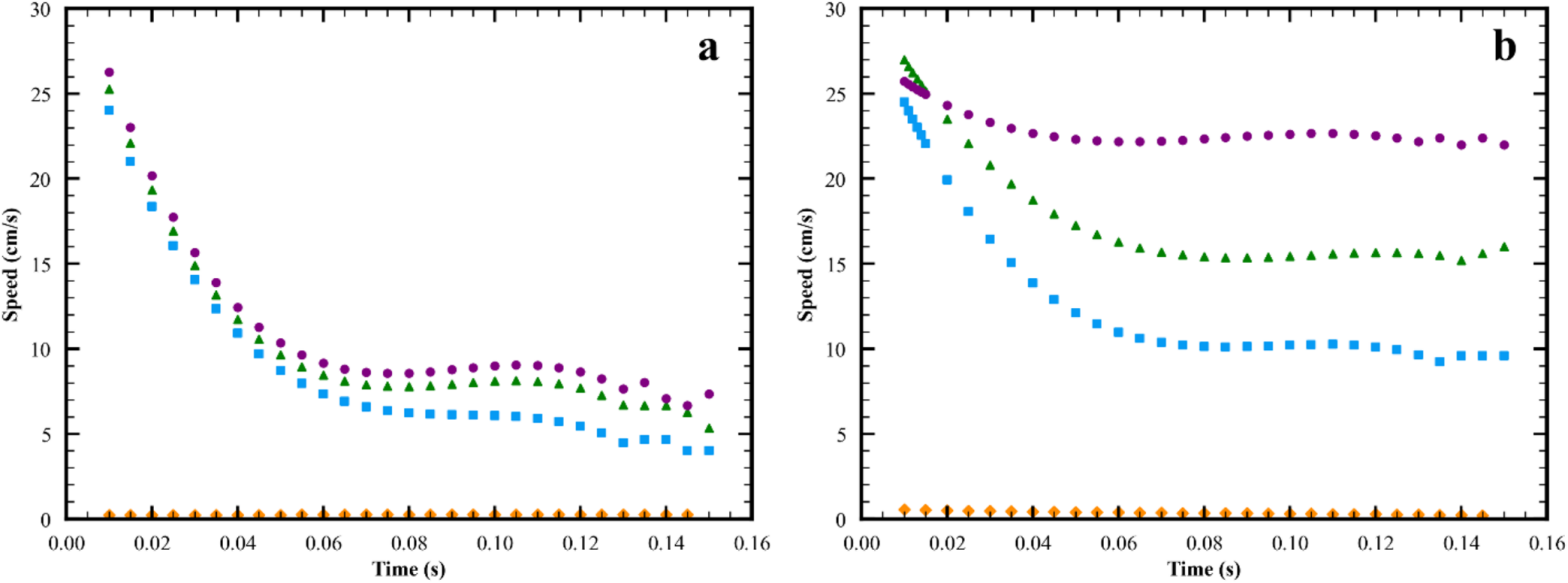
Changes in speed of surfactant front and S1 wave with subphase thickness at a viscosity of 1 mPa·s. (a) Surfactant front. (b) S1 peak. (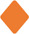, lubrication approximation; 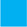, 1 mm; 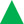, 2.4 mm; 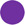, 5 mm) In (a), the speed of the lubrication approximation data ranges from 0.22 to 0.26 cm/s. In (b), the speed of the lubrication approximation data ranges from 0.56 to 0.19 cm/s. Parameters in lines 1, 2, 5, 11 in Table S2 and base case in Table S3.

With increasing viscosity, the increase of viscous stresses within the drop as it relaxes impacts the merging event, which depends on inertia to form the merging peak as the deposited drop relaxes. As seen in Figure S10 in SI, the timing of the formation and the speed of the merging peak change with viscosity; and for the highest viscosity examined, the merging peak never fully forms. As seen in Figure S12 in SI, for the thicknesses examined, subphase thickness again changes the timing of the formation and the speed of the merging peak.

While the speeds of the S wave and the surfactant front decrease with increasing viscosity, the decrease is different for the two features (see Figure 9). In all cases, the surfactant front moves more slowly than the S wave; and as a result, the condition for the splitting event is present at all viscosities. However, the difference in these speeds which sets the timing of the splitting event is complex and varies with viscosity. Thus, the timing of the plateau formation, the inflection point and the formation of the new maximum of the splitting event change in a complex way with increasing viscosity (see Figure S11 in SI). For the highest viscosity examined and in the limit of the lubrication approximation, these timing changes lead to the absence of the splitting event. As shown in Figure S15 in SI, the slowing of the S peak with increasing viscosity is consistent with experimental data obtained for the systems in lines 1, 4, and 7 in Table S1.

As seen in Figure 10, the speeds of the surfactant front and of S decrease with decreasing subphase thickness, with the effect on S being larger. In all cases, the surfactant front moves more slowly than the S wave. Accordingly, the splitting event does occur at all the thicknesses tested.

## 4 Conclusions

When inertia is significant–such as in the common case of Marangoni spreading on a water subphase thick enough to resist dewetting, the relaxation of a drop of surfactant solution deposited on a liquid subphase generates a wave packet which travels along the liquid surface. Unlike the regime usually examined in the literature of high viscosity and thin subphases where inertia is negligible and the lubrication approximation holds, this packet contains both waves whose surfaces are under the direct influence of the Marangoni stresses and waves which travel ahead of the surfactant front and are not directly influenced by Marangoni stresses. The initial conditions set by the relaxing surfactant-laden drop elongate the wave packet and cause it to contain more waves than would be present if the relaxing drop contained no surfactant. However, the hydrodynamics controlling the waves with no surfactant on their surfaces is similar to those controlling the propagation of capillary waves across the surface of the same subphase where the waves were generated by a surfactant-free drop.

Simulations and experiments demonstrate for the first time the merging of distinct waves at early times after the deposition of a surfactant-laden drop, an event which does not occur when no surfactant is present in the deposited drop. They also reveal the importance of the proximity and relative speeds of the (S) wave and the surfactant front which vary throughout the spreading event. The waves that are near the surfactant front and therefore are directly influenced by Marangoni stresses, undergo a complex evolution with time. During the relaxation of the deposited drop, a wave is generated that has surfactant covering part of its surface (the S wave) which follows behind the evolving packet of waves which have no surfactant. In addition, during the drop relaxation, another (merging) wave (the M wave) is generated after the S wave due to the bending of the drop-air surface. This latter wave moves faster than the next surfactant-covered peak at larger radial position (S). The merging peak is directly exposed to a gradient in surface tension while the largest surface tension gradient, associated with the leading surfactant front, occurs at nearly the same position as the maximum of the S wave at these early times. Eventually, that merging peak runs into and merges with the S peak. The simulation, validated by its ability to match three key experimental observations (the merging event, the tracking of the surfactant front relative to the S peak, and tracking of all peaks with time), shows that after the merging event the surfactant front with its large surface tension gradient moves more slowly than the S peak. This difference in speed subsequently splits into a surfactant-covered peak and a new, surfactant free peak which spreads in a manner consistent with a capillary wave, as do all the other surfactant-free peaks.

When decreasing the influence of inertia and increasing influence of viscous stresses by either increasing viscosity of both the drop and subphase or by decreasing initial subphase thickness, all peak heights are diminished and all peak and surfactant front speeds are decreased. However, the speed of the surfactant front and the peaks are not affected in the same manner by these changes. The capillary waves in the wave packet continue to show speeds predicted by the dispersion curves for capillary waves spreading on a surfactant free surface, where no surfactant is introduced into the system at all. The increased viscous stresses and decreased inertia only qualitatively change the characteristics of the merging event, until the lubrication approximation limit is reached, whereupon we observe a single peak, with the maximum surface tension gradient lying roughly under the peak as previously reported in the literature.

Our understanding of the new features reported here in solutal Marangoni spreading would be enhanced by experimental characterization of features of the spreading (especially the splitting event which has thus far only been seen in simulation) on liquid subphases where inertia dominates. Slowing the spreading down, by either increasing the viscosity of the subphase or decreasing the subphase thickness, diminishes the amplitudes of all waves, making them harder to experimentally observe with sufficient signal-to-noise to draw clear conclusions about their shape. Instead, high-speed imaging methods need to be developed with the required signal-to-noise ratio to provide quantitative measurements of the wave shapes throughout the spreading event. With improvements in the signal-to-noise of the experiments, the search for additional splitting events at later times would be possible. In the work reported here, deposition of the surfactant drop was deliberately limited to zero impact velocity. Future work should increase the deposition speed of the drop to form a bridge to research in the literature on impact of surfactant laden drops on deep liquid pools with higher impact speeds and thus nonzero Weber numbers.^58,59^ Treatment of a surfactant system above the critical micelle concentration and with more complex adsorption kinetics would be very valuable to probe how the wave packet structure and evolution are effected by these features of the surfactant system.

## Supplementary Material



## Notes

Systems examined (S1); simulation model (S2); simulation convergence (S3); interface shape evolution with time plotted with surface tension and surface tension gradient (S4); spreading for system within the lubrication approximation (S5); experimental data on variety of systems (S6); impact of drop volume on merging event (S7); interface shapes, surfactant excess profile and flow fields for diffusion limited and adsorption limited surfactant kinetics (S8); early time behavior when there is no surfactant in the deposited drop (S9); time scaling (S10), and effects of viscosity and subphase thickness (S11) (DOCX)
