# Supplementary material for: Complex Wave Packet Dynamics Induced by Marangoni Stresses
Source: Ind Eng Chem Res. 2025 Jul 11;64(29):14721–32. doi: 10.1021/acs.iecr.5c02378 (PMC12291194; doi:10.1021/acs.iecr.5c02378)
Supplement: Supplementary file 1 [file ie5c02378_si_001.docx]

# **Supporting Information**

**Complex Wave Packet Dynamics Induced by Marangoni Stresses**

Ruofan Shi^1^, Vignesh Thammanna Gurumurthy^2^, Robert D. Tilton^1, 3^, Stephen Garoff^4*^

^1^Department of Chemical Engineering, Carnegie Mellon University, Pittsburgh, PA 15213, USA

^2^ Department of Mechanical Engineering, Indian Institute of Technology Tirupati, Andhra Pradesh - 517619, India

^3^Department of Biomedical Engineering, Carnegie Mellon University, Pittsburgh, PA 15213, USA

^4^Department of Physics, Carnegie Mellon University, Pittsburgh, PA 15213, USA

*****Corresponding author: Department of Physics, Carnegie Mellon University, 5000 Forbes Avenue, Pittsburgh, PA 15213, USA (412)-268-6877, sg2e@andrew.cmu.edu.

Keywords: Marangoni flow, Marangoni Spreading, Surfactant spreading, Capillary waves

**S1 Systems examined**

**Table S1** Systems examined in experiments.

|  | Material System Subphase Composition | Subphase and drop Viscosity[1]  (mPa$\cdot$s) | Spreading Parameter[2]  (σ_drop_-σ_subphase_) (mN/m) | Initial Subphase Thickness (mm) | Aspect Ratio  ($\in=\frac{H_{0}}{R_{0}}$) [3] | Reynolds Number* ($Re\equiv\frac{\in*\Delta S*\rho_{sub}*R_{0}}{\mu_{sub}^{2}}$) [3,4] |
| --- | --- | --- | --- | --- | --- | --- |
| 1 | Water | 1 | 35.5 | 2.4 | 0.76 | 96000 |
| 2 | Water | 1 | 35.5 | 2.1 | 0.62 | 84000 |
| 3 | Water | 1 | 35.5 | 1.8 | 0.49 | 71000 |
| 4 | 20%glycerin/80%water | 1.8 | 36.5 | 2.4 | 0.76 | 31000 |
| 5 | 20%glycerin/80%water | 1.8 | 36.5 | 2.1 | 0.62 | 27000 |
| 6 | 20%glycerin/80%water | 1.8 | 36.5 | 1.8 | 0.49 | 23000 |
| 7 | 50%glycerin/50%water | 6.0 | 37.0 | 2.4 | 0.76 | 2700 |
| 8 | 50%glycerin/50%water | 6.0 | 37.0 | 2.1 | 0.62 | 2300 |
| 9 | 50%glycerin/50%water | 6.0 | 37.0 | 1.8 | 0.49 | 2000 |
| 10 | 80%glycerin/20%water | 60. | 33.0 | 2.4 | 0.76 | 26 |

* Re assumes balance of Marangoni and viscous stresses as appropriate for convective events

Parameters held constant:

SDS concentration = 82 mM

drop volume = 6 μL

Petri dish diameter =14.5cm

**Table S2** Systems examined in simulation

|  | Subphase and drop viscosity (mPa s) | Subphase initial surface tension (mN/m) | Drop initial surface tension (mN/m) | Initial Drop Radius (mm) | Initial Subphase Thickness (mm) | Aspect Ratio ($\in=\frac{H_{0}}{R_{0}}$) | Reynolds Number ($Re=\frac{\in*\Delta S*\rho_{sub}*R_{0}}{\mu_{sub}^{2}}$) |
| --- | --- | --- | --- | --- | --- | --- | --- |
| 1. | 1 | 72.5 | 32.6 | 1.8 | 5.0 | 2.74 | 197000 |
| 2.* | 1 | 72.5 | 32.6 | 1.8 | 2.4 | 1.32 | 95000 |
| 3. | 1 | 72.5 | 32.6 | 1.8 | 2.1 | 1.15 | 83000 |
| 4. | 1 | 72.5 | 32.6 | 1.8 | 1.8 | 0.99 | 71000 |
| 5. | 1 | 72.5 | 32.6 | 1.8 | 1.0 | 0.55 | 39000 |
| 6. | 1 | 72.5 | 32.6 | 1.8 | 0.2 | 0.11 | 7900 |
| 7. | 6 | 72.5 | 32.6 | 1.8 | 2.4 | 1.32 | 2600 |
| 8. | 60.1 | 72.5 | 32.6 | 1.8 | 2.4 | 1.32 | 26 |
| 9. | 1 | 72.5 | 32.6 | 18 | 2.4 | 0.13 | 950000 |
| 10. | 1 | 72.5 | 32.6 | 0.9 | 2.4 | 2.64 | 47500 |
| 11. ** | 1410 | 72.5 | 32.6 | 1.8 | 0.2 | 0.11 | 0.0040 |

* Base Case

** Lubrication approximation

Other parameters held constant

spreading parameter = ΔS = 39.9 mN/m^2^

isotherm parameters: Γ_m_=1x10^-5^ mole/m^2^, K= 20 m^3^/mole

initial concentration in drop = 0.2 mole/m^3^

D_b_ = D_s_ = 10^-9^ m^2^/s

domain = 0.3 m (no reflected waves from boundary in time scale of the simulations)

All fluids are incompressible

**Table S3** Surfactant kinetic parameters used in simulations.

| Surfactant Type | Adsorption rate constant (k_a_, m^3^ mol^-1^ s^-1^) | Damkohler Number of the Second Kind* |
| --- | --- | --- |
| Base case | 1000 | 320 |
| Reaction limited | 0.1 | 0.032 |
| Diffusion limited | 10000 | 3200 |

All other parameters are for the base case, line 2, Table S-2.

*DaII = (adsorption rate)/( diffusion rate to the interface) = L_diff_ ^2^k_a_c_0_/D_b_ , k_a_ is adsorption rate constant, c_0_ is initial concentration, D_b_ is bulk diffusivity, and L_diff_ is an estimation of diffusion layer thickness = $\frac{\Gamma_{eq}}{c_{0}}$.

**S2 Simulation model**

*Governing equations*

We consider the surfactant-induced spreading of a soluble surfactant-laden drop on an uncontaminated subphase of thickness *H_0_*. Both the drop and the subphase are made of the same Newtonian liquid. We model only the flow inside the film and neglect the air above it since its viscosity is small. Under laminar, incompressible conditions, the flow inside the subphase is governed by,

$$\begin{aligned} \rho\frac{D\vec{u}}{Dt}= -\vec{\nabla}P+ \mu\nabla^{2}\vec{u}\boldsymbol{+}\rho\vec{g} \#(S1) \end{aligned}$$

$$\begin{aligned} \nabla\cdot\vec{u}\boldsymbol{=}0 \#\left( S2 \right) \end{aligned}$$

where $\vec{g}$ (= 9.81 m/s^2^) is the acceleration due to gravity, $\vec{u}$ and *P* are the velocity and pressure fields, respectively.

Since the surfactant is soluble, its transport occurs both on the free surface and in the bulk. The concentration of the surfactant *c* in the bulk is governed by the advection-diffusion equation:

$$\begin{aligned} \frac{\partial c}{\partial t}+\vec{u}\cdot\vec{\nabla c}=D_{b}\nabla^{2}c \#\left( S3 \right) \end{aligned}$$

where *D_b_* is the surfactant diffusivity in the bulk phase. Similarly, on the free surface, the transport of the surface excess of the surfactant $\Gamma$ follows the advection-diffusion equation:

$$\begin{aligned} \frac{\partial\Gamma}{\partial t}+\vec{\nabla_{s}}\cdot\left( \Gamma\vec{u} \right)=D_{s}\nabla_{s}^{2}\Gamma+J \#\left( S4 \right) \end{aligned}$$

Here, *D_s_* is the surfactant diffusivity at the free surface, and $\nabla_{s}$ the surface gradient operator. The source term *J* represents the net surfactant adsorption flux from the bulk to the free surface, and is given by,

$$\begin{aligned} J= k_{a}\left( \Gamma_{m}-\Gamma\right)-k_{d}\Gamma\#\left( S5 \right) \end{aligned}$$

Here, *k_a_*, and *k_d_* are the adsorption and desorption rate constant, respectively. The equilibrium surface excess concentration $\Gamma_{m}$ is related to the surfactant concentration in the bulk as

$$\begin{aligned} \Gamma=\Gamma_{m}\frac{Kc}{1+Kc} \#\left( S6 \right) \end{aligned}$$

where *K* (*=k_a_/k_d_*) is the equilibrium constant. The surface tension, σ, is related to the surface excess of the surfactant by,

$\begin{aligned} \sigma=\sigma_{0}+RT\Gamma_{m}ln\left( 1-\frac{\Gamma}{\Gamma_{m}} \right) \#\left( S7 \right) \end{aligned}$where $\sigma_{0}$ is the surface tension coefficient of the clean interface, *R* is the gas constant, and T is the ambient temperature.

*Initial and boundary conditions*

The drop is modelled as a bump at the center of the dish. Now the initial height profile is initialized as follows,

$$\begin{aligned} H\left( r \right)= \left\{ \begin{aligned} H_{0}\left( 0.05\left[ cos\left( \frac{\pi r}{R_{0}} \right)+1 \right]+1 \right), 0\leq r\leq R_{0} \\ H_{0}, R_{0}\leq r\leq R_{dish} \end{aligned} \right. \#\left( S8 \right) \end{aligned}$$

Here, *r* is the radial coordinate, $R_{0}$ is the radius of the deposited drop, and $R_{dish}$ is the radius of the dish containing the subphase. The initial profile of the surfactant concentration in the bulk is defined as follows:

$$\begin{aligned} c\left( r,z \right)=c_{0}A\left( r \right)B\left( r,z \right)\#\left( S9 \right) \end{aligned}$$

where $A\left( r \right)$ and $B(r,z)$ are two error functions for smoothing the transition from the drop to initially unperturbed subphase to avoid numerical difficulties, and $c_{0}$ is the initial surfactant concentration in the drop. Their definitions are as follows,

$$\begin{aligned} A\left( r \right)=\frac{1}{2}+\frac{1}{2}\mathrm{erf} \left[ a\left( R_{0}-r \right) \right], B\left( r,z \right)=\frac{1}{2}+\frac{1}{2}\mathrm{erf} \left[ b\left( z-LD\left( r \right) \right) \right]\#\left( S10 \right) \end{aligned}$$

Here, a and b are constants set to 5000. *LD(r)* is a function that describes the lower drop boundary, which is defined as follows,

$$\begin{aligned} LD\left( r \right)=\left\{ \begin{aligned} H_{0}-0.05H_{0}\left[ cos\left( \frac{\pi r}{R_{0}} \right)+1 \right], 0\leq r\leq R_{0} \\ H_{0}, R_{0}\leq r\leq R_{dish} \end{aligned} \right. \#\left( S11 \right) \end{aligned}$$

The initial surface excess concentration, which is assumed to be in equilibrium with the bulk concentration of the drop, is defined as,

$$\begin{aligned} \Gamma\left( r \right)=\Gamma_{m}\frac{Kc_{s}}{1+Kc_{s}}\#\left( S12 \right) \end{aligned}$$

where $c_{s}$ is the surface concentration.

The boundary conditions are axial symmetry at *r =0*, no-slip at the dish bottom, and Navier-slip at the right wall r= $R_{dish}$. At the free surface, we have the stress jump boundary condition:

$\vec{n}\cdot\left[ -P\vec{I}+\mu(\vec{\nabla}\vec{u}+\left( \vec{\nabla}\vec{u} \right)^{T} \right]= \sigma{(\vec{\nabla}}_{s}\cdot\vec{n})\vec{n}-\vec{\nabla}_{s}\sigma$ (S13)

Here, $\vec{n}$ is the unit normal to the free surface, and $\vec{I}$ is the identity tensor. The surfactant-induced surface tension gradient is what drives the flow, which is accounted in the stress condition in the tangential direction.

The above equations are solved numerically using the finite element based commercial software COMSOL 5.6® under axisymmetric condition *(r,z)*. The above mathematical model has already been validated in our previous work [4] on predicting the spreading of an insoluble surfactant monolayer on a thin film.[3]

**S3 Simulation Convergence**

The mesh convergence study was conducted in three different meshes with an element count of 226011 (Finer), 618478 (Extra Fine), 1916138 (Extremely Fine) where the maximum element size was 0.00738, 0.00343, and 0.00177 respectively. Figure 1 compares the surface profile and the surfactant surface excess concentration at t= 0.015 s. We find the variation in surface height and surfactant concentration between different meshes to be minimal. Hence, we have used fine mesh in all our simulations.


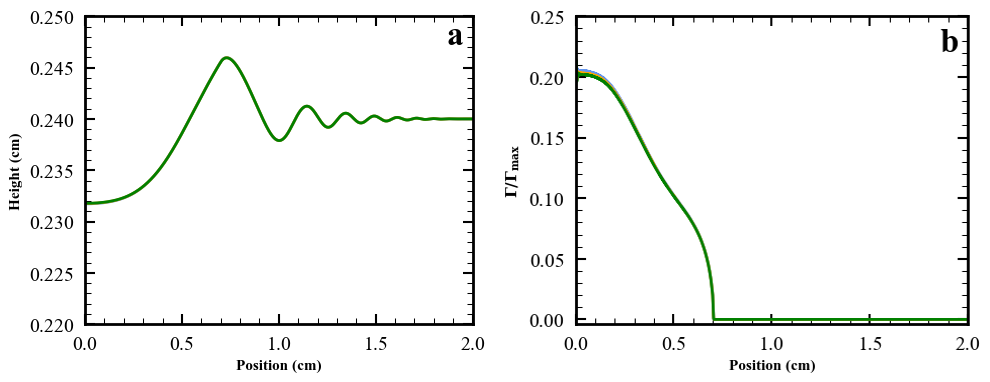


**Figure S1** Simulation results for different mesh sizes. (a) Surface height. (b) Surface excess concentration. ( ) fine mesh. ( ) finer mesh. ( ) extra fine mesh.

**S4 Interface shape evolution with time plotted with surface tension and surface tension gradient**

Figures S2 and S3 are the same as Figure 2 but with surface tension or surface tension gradients plotted instead of the surface excess.

**
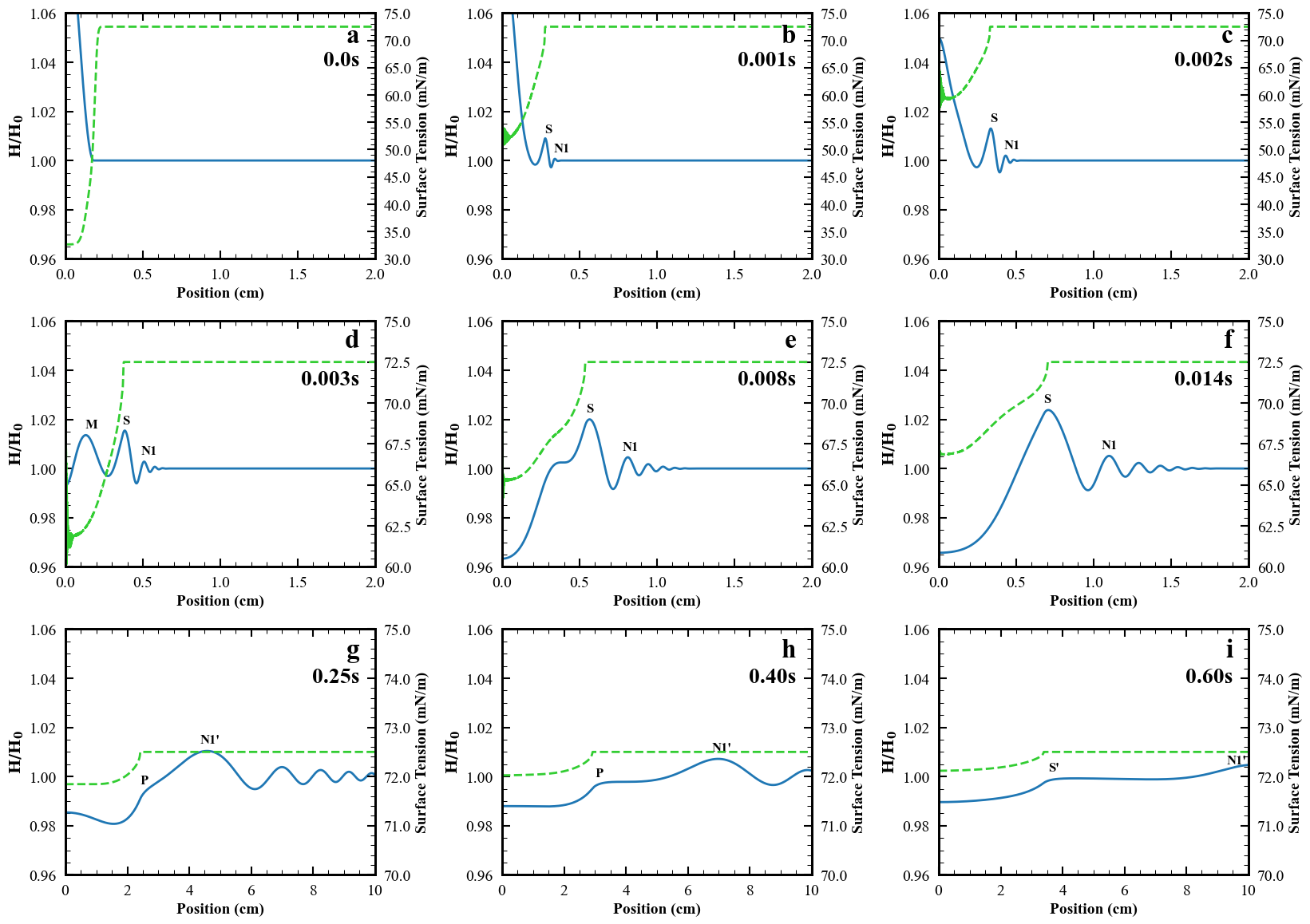
Figure S2** Spreading event for base case: soluble surfactant spreading on a 2.4 mm deep, 1 mPa s viscosity subphase (line 2 in Tables S2 and base case in Table S3). Figures include interface shape, blue solid line; surface tension, green dashed line. Panels a to c illustrate the relaxation of the deposited drop for (a) the initial condition, (b) t=0.001s, (c) t=0.002sec. Panels d to f show the merging event with (d) the merging peak, M, and the S peak before merging t=0.003sec, (e) the merging peak has been reduced to an inflection point during merging at t=0.008s, and (f) the S peak after merging at t=0.014 s. Panels g to i show the subsequent splitting event with (g) the S peak has been reduced to a the shoulder on the N1 peak at t=0.25s, (h) the shoulder has become stronger and is an inflection point in the interface shape at t=0.40s, and (i) the inflection has developed into a maximum marking a new peak S’ peak by t=0.6s. High frequency oscillations in surface tension near *r*=0 at early times arise from the shape used for the drop in the initial conditions (Eqn. S2 in SI) and have no effect on any behaviors discussed.

**
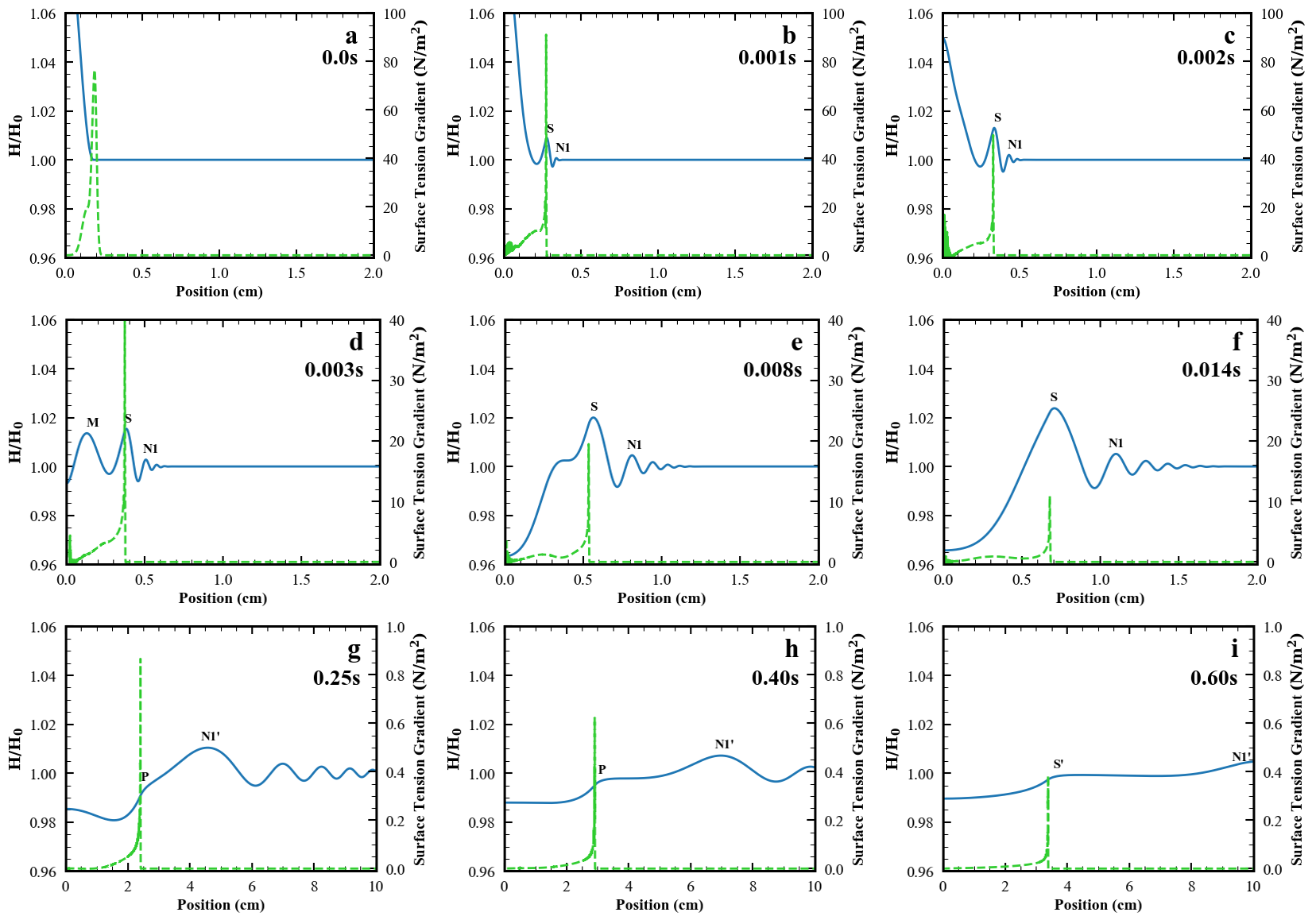
Figure S3** Spreading event for base case: soluble surfactant spreading on a 2.4 mm deep, 1 mPa s viscosity subphase (line 2 in Tables S2 and base case in Table S3). Figures include interface shape, blue solid line; surface tension gradient, green dashed line. Panels a to c illustrate the relaxation of the deposited drop for (a) the initial condition, (b) t=0.001s, (c) t=0.002sec. Panels d to f show the merging event with (d) the merging peak, M, and the S peak before merging t=0.003sec, (e) the merging peak has been reduced to an inflection point during merging at t=0.008s, and (f) the S peak after merging at t=0.014 s. Panels g to i show the subsequent splitting event with (g) the S peak has been reduced to a the shoulder on the N1 peak at t=0.25s, (h) the shoulder has become stronger and is an inflection point in the interface shape at t=0.40s, and (i) the inflection has developed into a maximum marking a new peak S’ peak by t=0.6s. High frequency oscillations in surface tension near *r*=0 arise from the shape used for the drop in the initial conditions (Eqn. S2 in SI) and have no effect on any behaviors discussed.

**S5 Spreading for system within the lubrication approximation**

Figure S4 shows the interface shape, surfactant excess, and velocity fields for a system that can be treated within the lubrication approximation (line 11 Table S2). All waves with no surfactant at their surfaces have been suppressed. A single wave with surfactant on its surface remains. The merging and splitting events have also been suppressed.

**
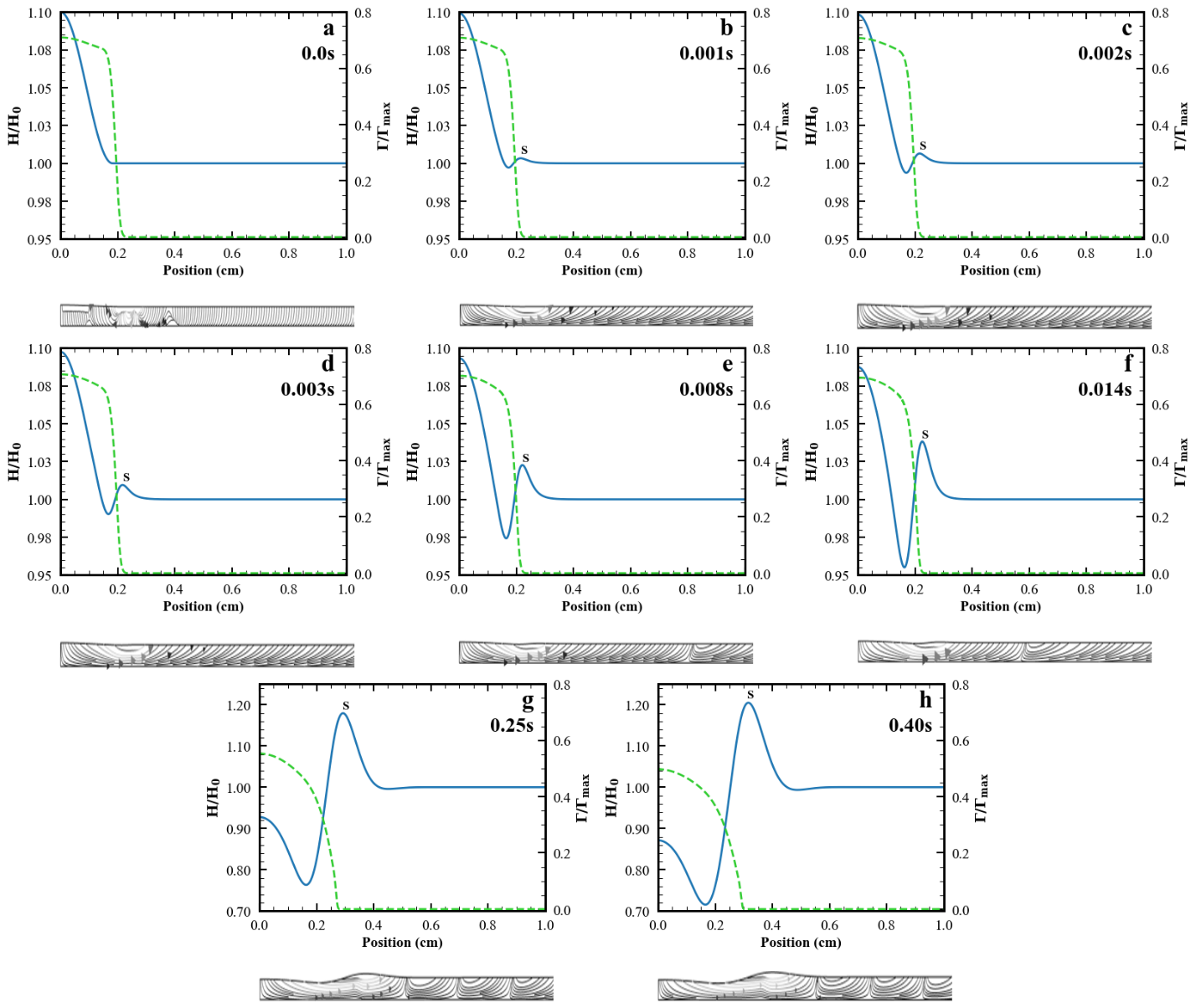
Figure S4** Spreading event for lubrication approximation case: soluble surfactant spreading on a 0.2 mm deep, 1410 mPa s viscosity subphase (line 11 on Table S2 and base case in Table S3). Figures include interface shape, blue solid line; surfactant surface excess concentration, green dashed line; and flow field, black. Panels a to c illustrate the relaxation of the deposited drop for (a) the initial condition, (b) t=0.001s, (c) t=0.002sec. Panels d to f show times when merging event would have occurred in base case (d) t=0.003sec, (e) t=0.008sec, and (f) after merging would have been completed, t=0.014 sec. Panels g and h show times when splitting event waould have occurred in base case (g) when the shoulder would have formed, t=0.25sec, (h) when the inflection point during splitting would have occurred, t=0.40sec.

**S6 Experimental data on variety of systems**

Figure S5 shows the experimental position vs time for systems in lines 4, 5, 6, 7, 8 in Table S1. These data are very similar to the base case experimental and simulation systems shown in Figure 5.


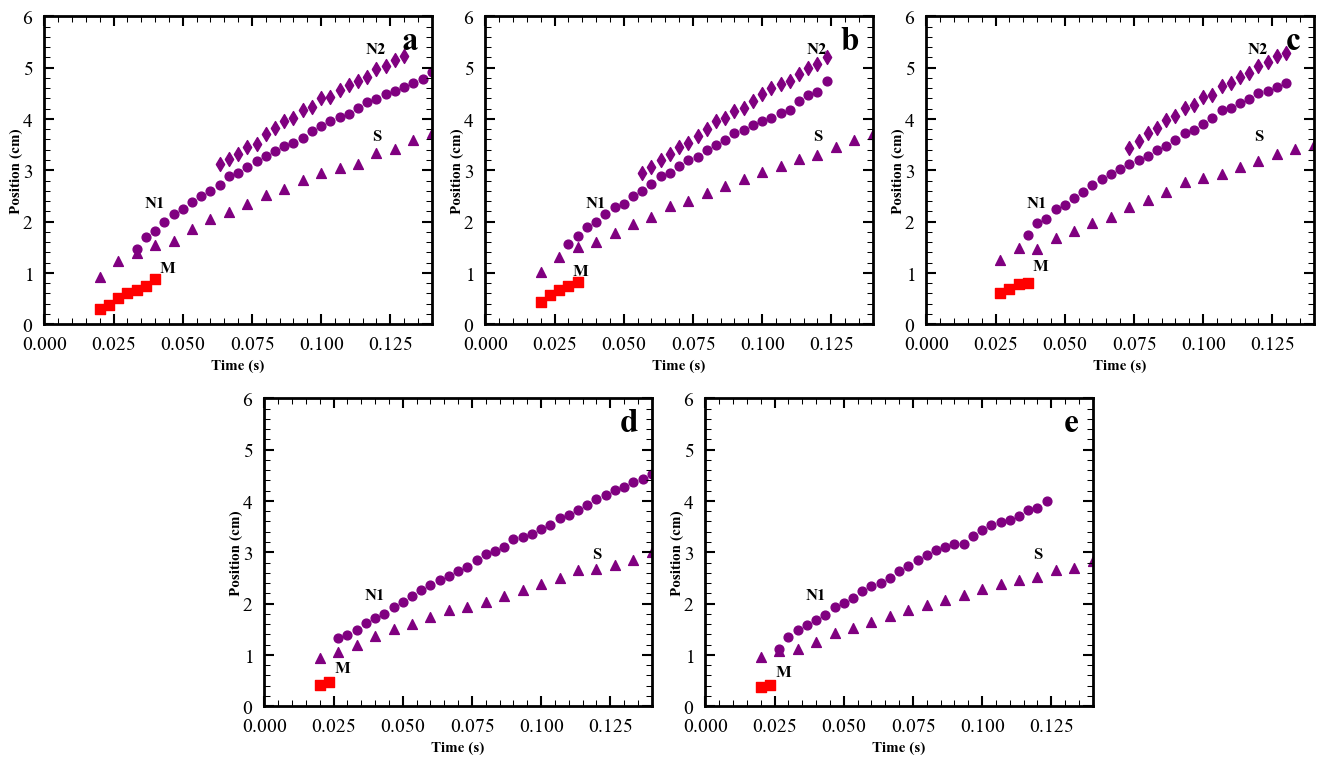


**Figure S5** Experimental data for position vs time for various systems. ■, M. ▲, S. •, N1. ♦, N2. (a) Viscosity 1.8 mPa s, subphase thickness 2.4 mm; line 4 in Table S1. (b) Viscosity 1.8 mPa s, subphase thickness 2.1 mm; line 5 in Table S1. (c) Viscosity 1.8 mPa s, subphase thickness 1.8 mm; line 6 in Table S1. (d) Viscosity 6 mPa s, subphase thickness 2.4 mm; line 7 in Table S1. (e) Viscosity 6 mPa s, subphase thickness 2.1 mm; line 8 in Table S1.

**S7 Impact of drop volume on merging event**

Figure S6 shows that even when the drop size is reduced by a factor of twenty below our base case, the merging event still occurs.

**
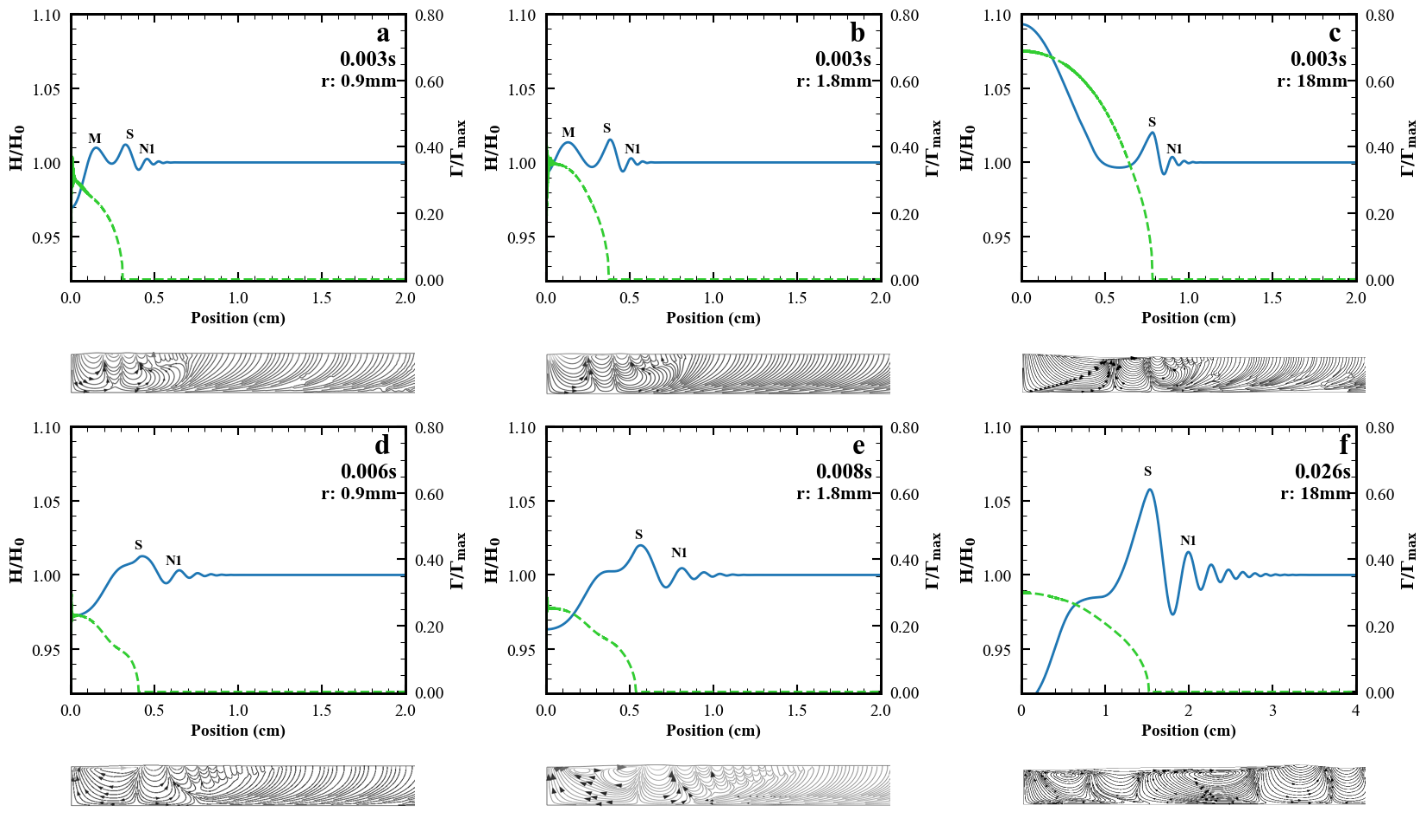
Figure S6** Interface shape, surface converage, and flow field during merging event as the drop size is varied a 2.4 mm deep, 1 mPa s viscosity subphase. . Figure includes interface shape, blue solid line; surfactant concentration, green dashed line; and flow field, black. Initial drop radius varies from 0.9 mm to 1.8 mm to 18mm from left to right. Panels a to c, before merging (all at t=0.003). Panels d to f, inflection point formation (d, t=0.006; e, t=0.008; f, t=0.026). Parameters as in lines 2, 9, 10 Table S2 and base case in Table S3.

**S8 Interface shapes, surfactant excess profile and flow fields for diffusion limited and adsorption limited surfactant kinetics**

Spreading is only qualitatively changed by adsorption kinetics even at the limits of adsorption and diffusion limited transport. As an example, Figure S7, shows the interface shape and surfactant excess during the merging event.

**
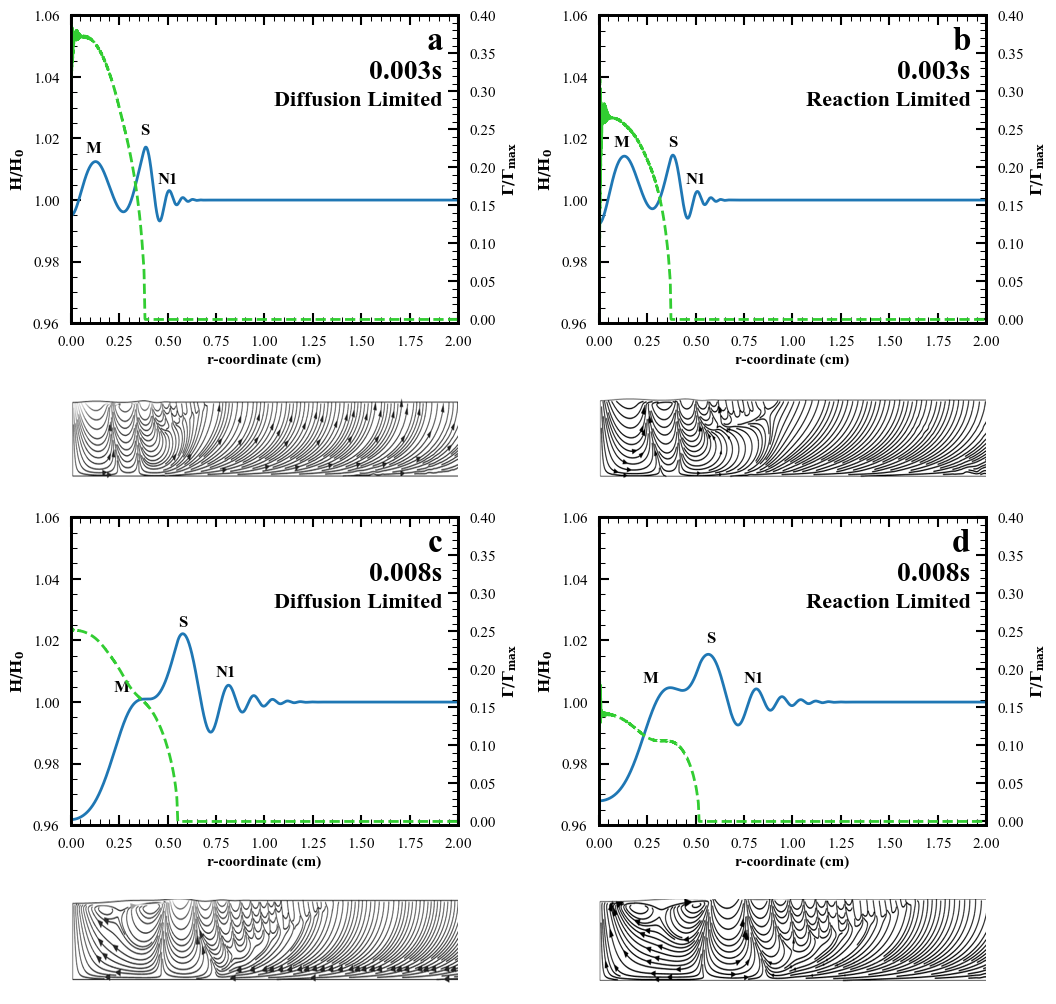
Figure S7** Spreading for different adsorption kinetics on a 2.4 mm deep, 1 mPa s viscosity subphase. Figures include interface shape, blue solid line; surface tension, green dashed line; flow fields, black. (a) Before merging, t=0.003 s (k_a_=10000 m^3^ mol^-1^ s^-1^, DaII= 3200). (b) Before merging, t=0.003 s (k_a_=0.1 m^3^ mol^-1^ s^-1^, DaII=0.032). (c) Inflection point, t=0.008 (k_a_=10000 m^3^ mol^-1^ s^-1^, DaII=3200). (d) Inflection point, t=0.008 (k_a_=0.1 m^3^ mol^-1^ s^-1^, DaII=0.032). Parameters found in line2 in Table S2 and 2^nd^ and 3^rd^ lines in Table S3.

**S9 Early time behavior when there is no surfactant in the deposited drop**

Figure S8 shows the early time behavior of the interface shape when there is no surfactant in the deposited drop. There is no equivalent of the merging event which appears when there is surfactant in the drop. Waves form at small r, joining the wavepacket which propagates outward with time.


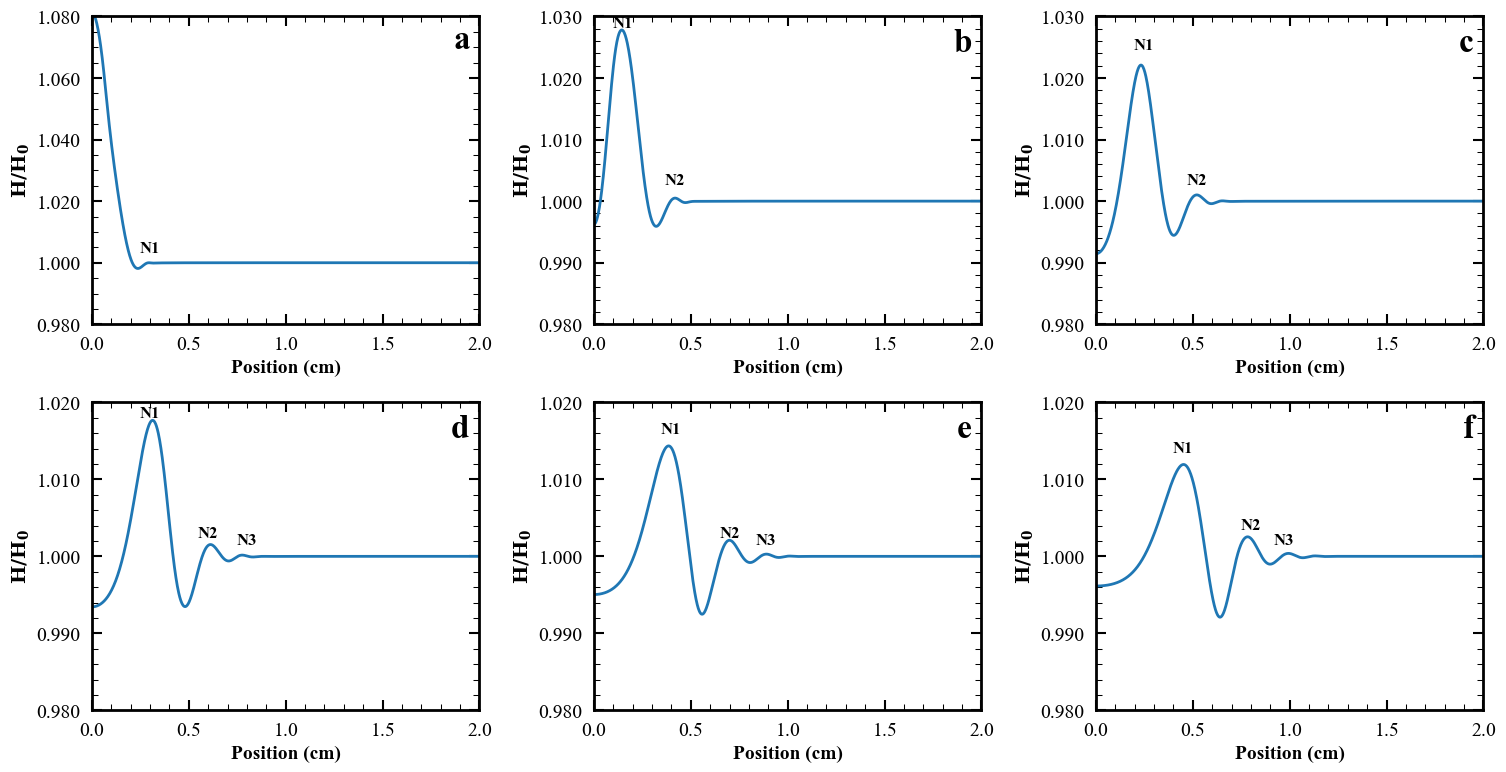


**Figure S8** Interface shape at early times with no surfactant in the deposited drop. All other parameters the same as in Figure 2.

**S10 Time scaling**

Since all cases treated in the present work are outside the lubrication approximation, the features of the spreading event are not expected to occur at the same scaled time predicted for the lubrication approximation ( $t_{c}=\frac{\mu R_{0}^{2}}{SH_{0}}$) [3] as parameters change. None of the results in this paper collapse using the lubrication approximation time scaling. Figure S9 shows one example of that failure.


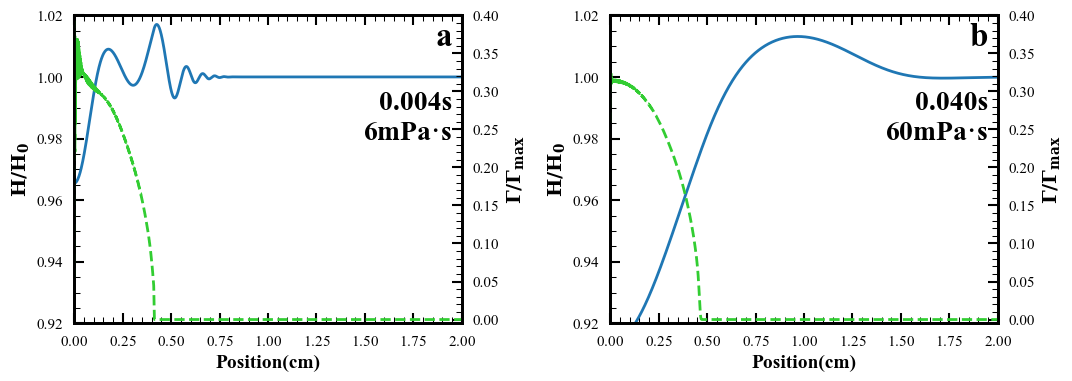


**Figure S9** Interface shape of two different viscosity subphases at fixed 2.4 mm thickness shown at the same scaled time = 20. (a) Interface shape and surface excess of a 6 mPa$\cdot$s subphase at t=0.004 sec. (line 7 Table S-2) (b) Interface shape of a 60 mPa$\cdot$s subphase at t=0.04 sec. Parameters found in lines 7, 8 in Table S2 and base case in Table S3.

**S11 Effects of viscosity and subphase thickness**

As viscosity increases, the number of peaks with no surfactant on their surfaces decreases, and their heights decrease. (See Figure S10 for data during the merging event and Figure S11 for data during the splitting event.) As seen in Figure S10, the timing of the waves involved in the merging event changes with viscosity and merging does not occur for 60 mPa⋅s. Similarly as seen in Figure S11, the timing of the surfactant front separating from the S peak also is affected by viscosity, changing the splittng event. The splitting event does not occur for 60 mPa⋅s.

**
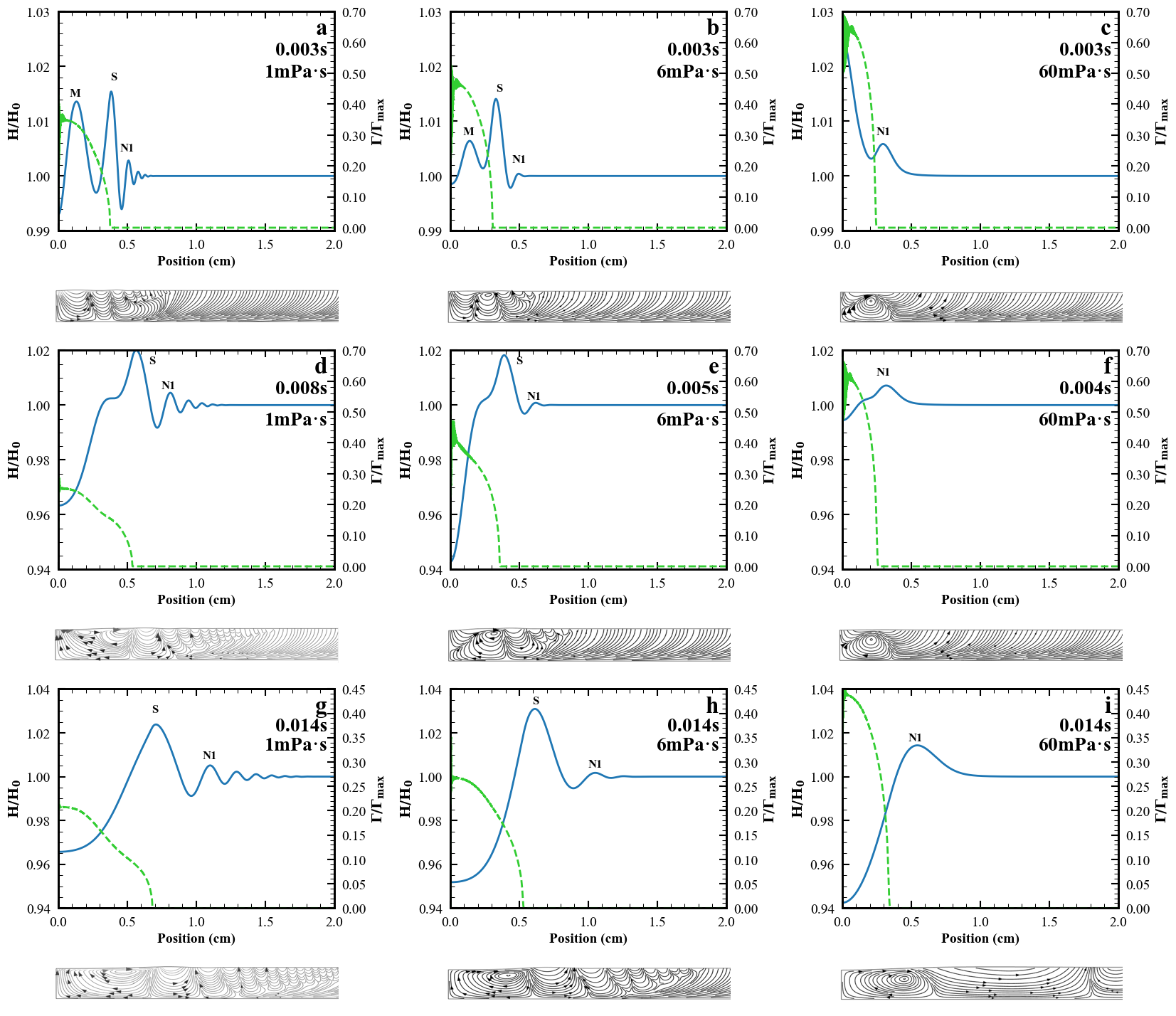
Figure S10** Interface shape, surface converage, and flow field during merging event as the viscosity is varied at fixed 2.4mm subphase thickness. Figure includes interface shape, blue solid line; surfactant concentration, green dashed line; and flow field, black. Viscosity varies from 1 mPa⋅s to 6 mPa⋅s to 60 mPa⋅s from left to right. Panels a to c, before merging. Panels d to f, inflection point formation. Panels g to i, after merging. Parameters found in lines 2, 7, 8 in Table S2 and base case in Table S3.

**
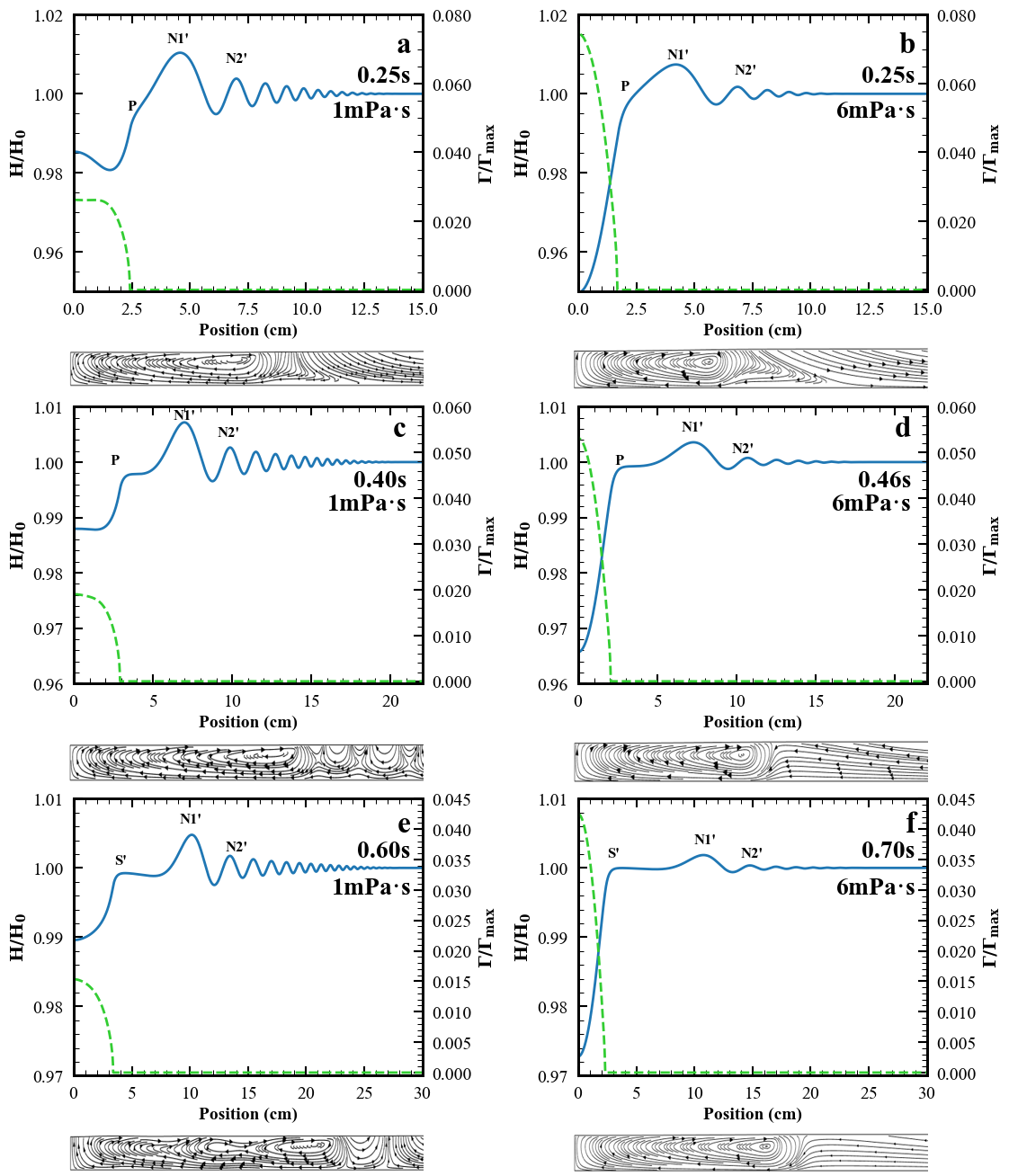
Figure S11** Interface shape, surface converage, and flow field during splitting event as the viscosity is varied at fixed 2.4 mm subphase thickness. Figure includes interface shape, blue solid line, surfactant surface concentration, green dashed line; and flow field, black. Viscosity varies from 1 mPa⋅s to 6 mPa⋅s from left to right. Panels a to b, Marangoni plateau formed. Panels c to d, inflection point formed. Panels e to f, new S’ peak formed. Note: The spitting event does not occur for 60 mPa⋅s . Parameters found in lines 2, 7 in Table S2 and base case in Table S3.

As subphase thickness decreases, the number of peaks with no surfactant on their surfaces and their heights slightly decrease. (See Figure S12.) The timing of the waves involved in the merging event changes with subphase thickness.

**
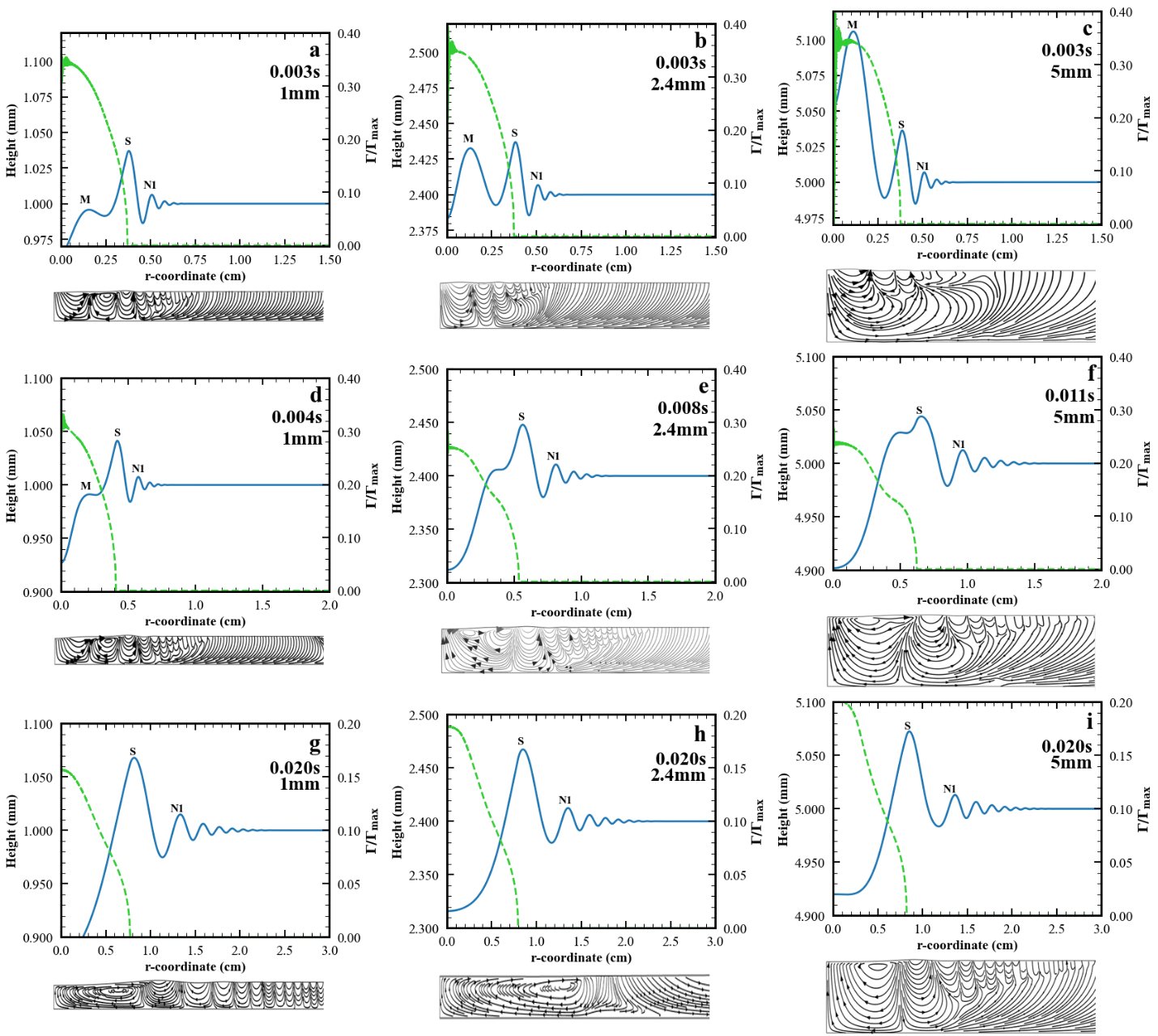
**

**Figure S12** Interface shape, surface converage, and flow field during merging event as the subphase thickness is varied at fixed 1 mPa⋅s subphase viscosity. Figure includes interface shape, blue solid line (height not normalized); surfactant concentration, green dashed line; and flow field, black. Subphase thickness varies from 1 mm to 2.4mm to 5 mm from left to right. Panels a to c, before merging. Panels d to f, inflection point formation. Panels g to i, after merging. Parameters found in lines 1, 2, 5 in Table S2 and base case in Table S3.

Experimental data shows the same trend with increasing viscosity for waves for no surfactant on their surface as simulation: the speed of the peaks decreases as the viscosity increases. (See Figure S13.)


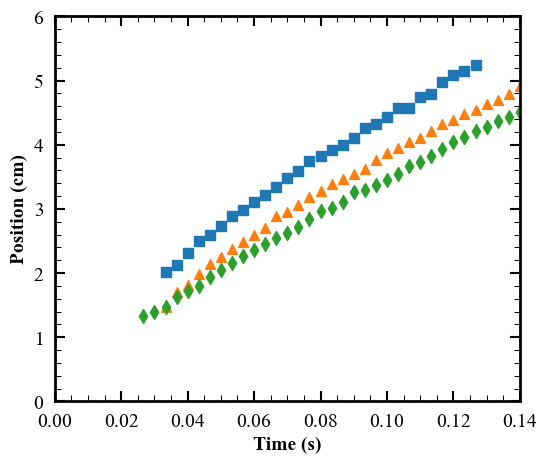


**Figure S13** Example of experimental data for position vs time for systems at fixed subphase thickness (2.4 mm) but varying viscosity for N1 peak. (blue squares, ■) 1 mPa⋅s, line 1 in Table S1. (orange triangle, ▲) 1.8 mPa⋅s, line 4 in Table S1. (green diamonds, ♦) 6.0 mPa⋅s, line 7 in Table S1.

Experimental data shows the same trend with decreasing subphase thickness for waves for no surfactant on their surface as simulation: wave change little over the range of thickness examined. (See Figure S14.)


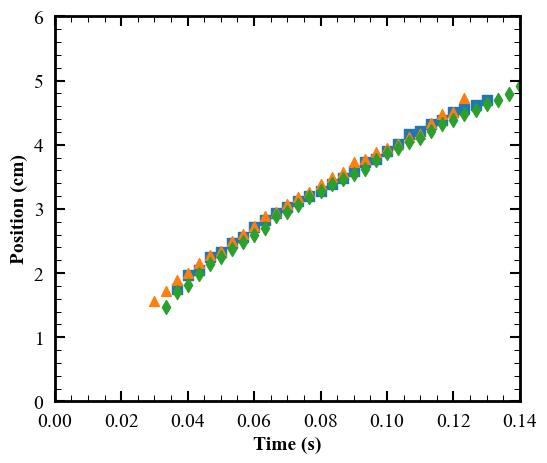


**Figure S14** Example of experimental data for position vs time for systems at fixed subphase viscosity (1.8 mPa s) but varying subphase thickness for N1 peak. (blue squares, ■) 1.8 mm, line 6 in Table S1. (orange triangles, ▲) 2.1 mm, line 5 in Table S1. (green diamonds, ♦) 2.4 mm, line 4 in Table S1.

Figures S10, S11 and S12 show that, in simulation, the speed of the S peak decreases with increasing viscosity. The experimental data shows the same trend. (See Figure S15.)


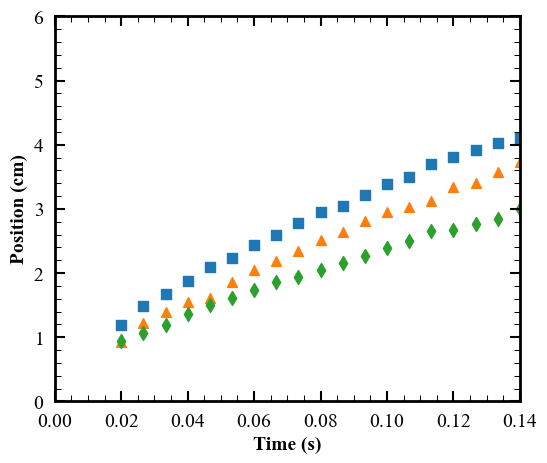


**Figure S15** Example of experimental data for position vs time for systems at fixed subphase thickness (2.4 mm) but varying viscosity for S peak. (blue squares, ■) 1 mPa⋅s, line 1 in Table S1. (orange triangle, ▲) 1.8 mPa⋅s, line 4 in Table S1. (green diamonds, ♦) 6.0 mPa⋅s, line 7 in Table S1.

**References**

[1] K. Takamura, H. Fischer, N.R. Morrow, Physical properties of aqueous glycerol solutions, J Pet Sci Eng 98–99 (2012) 50–60. https://doi.org/10.1016/j.petrol.2012.09.003.

[2] H. Khan, J.M. Seddon, R. V Law, N.J. Brooks, E. Robles, J.T. Cabral, O. Ces, Effect of glycerol with sodium chloride on the Krafft point of sodium dodecyl sulfate using surface tension, J Colloid Interface Sci 538 (2019) 75–82. https://doi.org/10.1016/j.jcis.2018.11.021.

[3] D.P. Gaver, J.B. Grotberg, The dynamics of a localized surfactant on a thin film, J Fluid Mech 213 (1990) 127. https://doi.org/10.1017/S0022112090002257.

[4] M.L. Sauleda, T.-L. Hsieh, W. Xu, R.D. Tilton, S. Garoff, Surfactant spreading on a deep subphase: Coupling of Marangoni flow and capillary waves, J Colloid Interface Sci 614 (2022) 511–521. https://doi.org/10.1016/j.jcis.2022.01.142.
